# Inulin‐Based Oral Chemotherapy Modulates Gut Microbiota and Immune Microenvironment through Inhibition of Neutrophil Extracellular Trap Formation for Improving Cancer Therapy

**DOI:** 10.1002/advs.202514318

**Published:** 2026-01-29

**Authors:** Zhenhao Li, Jiaqiang Jiang, Yuping Zhou, Xinyuan Mao, Haotian Wen, Zhian Chen, Yutong Wang, Cong Huang, Yanfeng Hu

**Affiliations:** ^1^ Department of General Surgery Guangdong Provincial Key Laboratory of Precision Medicine for Gastrointestinal Tumors Nanfang Hospital Southern Medical University Guangzhou China; ^2^ South China University of Technology School of medicine Guangzhou China; ^3^ The First Affiliated Hospital of Shantou University Medical College Shantou China

**Keywords:** chemoimmunotherapy, colorectal cancer, gut microbiota, inulin, neutrophil extracellular trap

## Abstract

Despite the potential of chemoimmunotherapy against colorectal cancer (CRC) development, adverse reactions and drug resistance hinder its clinical uptake. Dysbiosis of the gut microbiota and neutrophil extracellular traps (NETs) formation are implicated in the increasing prevalence of chemoimmunotherapy resistance, which will need to be regulated to enable effective chemotherapy against CRC. To improve CRC therapy, inulin‐based oral chemotherapy microspheres (ZIF‐8@OXA@Inulin) are constructed. Oral inulin‐based microspheres specifically adapt to the digestive system, which accurately release drugs in response to pH variations in the gastrointestinal tract. Moreover, ZIF‐8@OXA@Inulin significantly enhances the intratumoral concentration and retention time of chemotherapeutic agents in an orthotopic colon tumor model, thereby further promoting pyroptosis. Additionally, ZIF‐8@OXA@Inulin microspheres reshape the gut microbiota by increasing the abundance of beneficial bacterial genera (*Alistipes*, *Lactobacillus*, *Enterorhabdus*, and *Turicibacter*) and suppressing the formation of NETs induced by chemotherapy‐associated side effects, ultimately facilitating T cell infiltration within the tumor microenvironment. ZIF‐8@OXA@Inulin microspheres not only demonstrate excellent anti‐tumor performance and antimetastatic effect but also exhibit adjuvant effects to immunotherapy agents. Collectively, the oral inulin‐based microspheres (ZIF‐8@OXA@Inulin) are capable of exhibiting anti‐tumor activities while promoting chemotherapy and immunotherapy effects. Therefore, they show substantial application potential for CRC therapy.

## Graphical abstract

Schematic representation of ZIF‐8@OXA@inulin for cancer immunotherapy. Synthesis of ZIF‐8@OXA@inulin. Augmentation of pyroptosis, regulation of gut microbiota, and inhibition of NETs formation by ZIF‐8@OXA@inulin‐mediated comprehensive strategy for antitumor immune response.

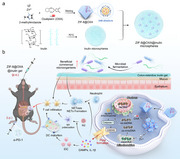

## 1 Introduction

The proposed mechanisms underlying chemoimmunotherapy include the inhibition of immunosuppressive cells, activation of effector cells, enhancement of immunogenicity, and increased T‐cell infiltration [1, 2]. However, immunotherapy alone exhibits limited efficacy in colorectal cancer (CRC), primarily because of poor immunogenicity and an immunosuppressive tumor microenvironment (iTME) [3]. Neutrophils are essential for the regulation and orchestration of adaptive immune responses and contribute to various immune functions, including phagocytosis and the formation and release of extracellular traps, known as neutrophil extracellular traps (NETs) [4, 5]. NETs comprise decondensed chromatin fibers, including DNA, histones, and other proteins released by activated neutrophils. These structures significantly influence the immune landscape of the tumor microenvironment (TME), affecting both local immune responses and tumor progression [5, 6]. Consequently, there has been an increasing focus on the role of neutrophils and NETs in shaping the TME. Recent studies have indicated that NETs play a significant role in tumor recurrence, metastasis, and therapeutic resistance [5, 6, 7]. Moreover, anti‐NET therapy has the potential to exhibit synergistic antitumor effects when combined with chemotherapy. Thus, using various strategies to inhibit NET formation, such as the application of DNase I [8, 9, 10] and modulation of the gut microbiota [11, 12], is crucial for enhancing the efficacy of chemoimmunotherapy.

Gut microbial dysbiosis is common in chemoimmunotherapy, and studies have demonstrated a correlation between gut microbial dysbiosis and poor treatment outcomes [13, 14]. Thus, preventing chemoimmunotherapy‐induced microbial dysbiosis could enhance the synergistic effects of the treatment [15]. Oral drug delivery systems targeting the colon have gained attention as effective strategies for treating gastrointestinal (GI) disorders and enhancing therapeutic outcomes [16, 17, 18, 19, 20]. However, the acidic environment of the stomach and enzymes in GI, such as pepsin, trypsin, and glucosidase, present substantial obstacles to the successful delivery of drugs to the colon. Inulin, a formulation ingredient approved by the Food and Drug Administration (FDA) for oral drug delivery, has shown promise in protecting drugs from acidic and enzymatic environments in the GI tract [21], further enhancing drug retention and accumulation in the colon [22, 23]. Moreover, accumulating evidence suggests that inulin undergoes targeted degradation by beneficial bacteria in the colon, which exerts probiotic‐like effects by modulating the intestinal microenvironment [22]. These effects include the suppression of harmful bacteria, promotion of beneficial bacteria, and support for microbiota restoration [24]. Furthermore, these beneficial bacteria have been shown to trigger antitumor immune responses and inhibit the formation of NETs [12, 25], thereby improving the efficacy of chemoimmunotherapy and survival rates in patients with cancer.

In this study, we developed an orally administered inulin microsphere containing ZIF‐8 nanoparticles (NPs) loaded with the chemotherapeutic drug oxaliplatin (OXA) (ZIF‐8@OXA@inulin). As shown in the graphical abstract, for CRC, which commonly exhibits an immunosuppressive tumor microenvironment, ZIF‐8@OXA@inulin achieved: i) Controlled release in the GI tract. We developed an oral inulin‐based microsphere specifically adapted to the digestive system. This intelligently released drugs in response to pH variations in the GI tract. ii) Colon targeting and retention of the drug. We established a microbiota‐targeted drug delivery microsphere, ZIF‐8@OXA@inulin, which demonstrated high efficiency in targeting CRC and ensured prolonged retention in the colon of mice. This platform increased the blood concentration of chemotherapeutic agents, ensuring targeted delivery to CRC sites. iii) TME‐programmed therapeutics. ZIF‐8@OXA@inulin effectively modulates gut microbiota by enhancing the abundance of beneficial bacterial communities and preventing NETs formation, thereby promoting T‐cell infiltration within the TME. iv) Chemoimmunotherapy enhancement. ZIF‐8@OXA@inulin effectively enhances chemoimmunotherapy by inducing tumor cell pyroptosis, inhibiting NETs formation, and promoting beneficial bacterial colonization. In an orthotopic colon tumor model, when combined with an immunotherapeutic agent, the anti‐programmed death‐1 antibody (αPD‐1), ZIF‐8@OXA@Inulin exhibited excellent antitumor effects. v) Biosafety. ZIF‐8@OXA@inulin demonstrated excellent biocompatibility and holds significant potential for future clinical translation and CRC treatment. In summary, ZIF‐8@OXA@inulin exhibited superior chemotherapy and promoted immunotherapy by regulating the gut microbiota and inhibiting NETs formation, providing novel insights into CRC treatment.

## 2 Results

### 2.1 Design, Synthesis, and Characterization of ZIF‐8@OXA@Inulin Microspheres

**FIGURE 1:**
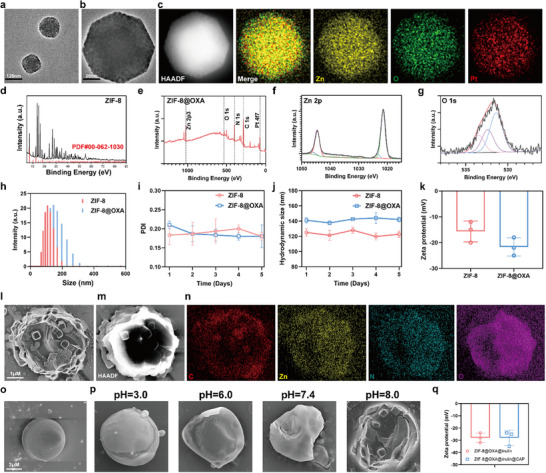
Design, synthesis, and characterization of ZIF‐8@OXA@inulin. (a) TEM images of ZIF‐8@OXA NPs, scale bar: 125 nm. (b) Enlarged TEM images of ZIF‐8@OXA NPs, scale bar: 25 nm. (c) High‐angle annular dark‐field TEM image and elemental mapping of ZIF‐8@OXA NPs. (d) XRD pattern of ZIF‐8@OXA NPs. (e) XPS of ZIF‐8 and ZIF‐8@OXA NPs. (f, g) High‐resolution XPS scans of Zn 2p (f) and O 1s (g) in ZIF‐8@OXA NPs. (h) Hydrodynamic size distribution of ZIF‐8 and ZIF‐8@OXA NPs. (i) PDI of ZIF‐8 and ZIF‐8@OXA NPs in different times. (j) Hydrodynamic size distribution of ZIF‐8 and ZIF‐8@OXA NPs in different times. (k) Zeta potential (mV) of ZIF‐8 and ZIF‐8@OXA NPs (n = 3). (l) SEM image of ZIF‐8@OXA@inulin microspheres. (m, n) HAADF‐STEM image and elemental mapping of ZIF‐8@OXA@inulin microspheres. (o) SEM image of ZIF‐8@OXA@inulin@CAP microspheres. (p) The degradation ability of ZIF‐8@OXA@inulin@CAP microspheres. (q) Zeta potential (mV) of microspheres (n = 3).

First, ZIF‐8@OXA NPs were prepared by assembling Zn^2+^ with organic 2‐methylimidazole in a methanol solution, and OXA was incorporated simultaneously [26, 27, 28]. To improve biocompatibility, we surface‐modified ZIF‐8 and ZIF‐8 loaded with oxaliplatin using Pluronic F127 and refer to these formulations as ZIF‐8 and ZIF‐8@OXA NPs, respectively (Figure S1). As shown in Figure 1a,b, ZIF‐8@OXA NPs exhibited a crystal structure with a particle size of approximately 130 nm. This observation demonstrates the successful addition of OXA, which did not affect the original structure of ZIF‐8 NPs. The presence of Pt in the elemental mapping (Figure 1c) further confirmed the successful incorporation of OXA into ZIF‐8 NP_S_. In addition, consistent with the HRTEM results, the X‐ray diffraction (XRD) patterns of the as‐prepared ZIF‐8 NPs were similar (Figure 1d) [26, 27, 28]. The Pt content within the ZIF‐8@OXA NPs was detected based on inductively coupled plasma‐mass spectrometry (ICP‐MS), and the loading efficiency of OXA was approximately 8.8%. X‐ray photoelectron spectroscopy (XPS) was used to determine the sample compositions. Among the XPS high‐resolution scans of the Zn 2p and O 1s peaks, no obvious shift was observed for the ZlF‐8 and ZIF‐8@OXA NPs, indicating a negligible effect of OXA on the coordination of Zn ions and 2‐methylimidazole (Figure 1e–g). The hydrodynamic sizes of the different NPs were confirmed using dynamic light scattering (DLS) (Figure 1h). In addition, ZIF‐8 NPs and ZIF‐8@OXA NPs exhibited a polydispersity index (PDI) of 0.18 ± 0.01 and 0.17 ± 0.03 in PBS, respectively (Figure 1i), and exhibited hydrodynamic size distributions of 125.43 ± 2.43 and 142.19 ± 3.95 nm in PBS after 5 days, respectively (Figure 1j). Moreover, Figure 1k confirms the negative charge of the ZIF‐8@OXA NPs, which were equipped with circulation stability. These results demonstrate the successful synthesis of ZIF‐8@OXA NPs.

Inulin was selected as the polysaccharide matrix for hydrogel fabrication because of its favorable physicochemical and biological attributes. Its excellent biocompatibility ensures suitability for biomedical use, and the hydroxyl‑rich backbone enables extensive cross‑linking that produces mechanically robust hydrogels with strong mucoadhesion and prolonged colonic residence. As a prebiotic, inulin is cleaved by colonic inulinase to short‑chain metabolites that selectively stimulate *Bifidobacterium* and *Lactobacillus* growth, thereby modulating the gut microbiota. Harnessing these characteristics, we coated ZIF‐8@OXA NPs with inulin to protect the payload and achieve colorectum‑specific release. Initially, we enveloped ZIF‐8@OXA NPs with an inulin shell and then added a cellulose acetate phthalate (CAP) overlayer to protect the resulting microspheres from gastric acid. This sequential encapsulation yielded stable ZIF‐8@OXA@inulin microspheres. The ZIF‐8@OXA@inulin microspheres were characterized using scanning electron microscopy (SEM), demonstrating uniformly dispersible microspheres in solutions and spheroidal morphology (Figure 1l). High‐angle annular dark‐field scanning TEM (HAADF‐STEM)‐based elemental mapping and TEM‐EDS (energy‐dispersive X‐ray spectroscopy) revealed that the as‐prepared ZIF‐8@OXA@inulin microspheres contained C, N, O, and Zn elements, which were homogeneously distributed in the ZIF‐8@OXA@inulin microspheres (Figure 1m,n). Furthermore, to prevent the microspheres from being degraded by gastric acid, we coated them with CAP, thereby ultimately synthesizing ZIF‐8@OXA@inulin@CAP microspheres. The ZIF‐8@OXA@inulin@CAP microspheres were characterized by SEM, which revealed uniformly dispersible microspheres in solutions (pH 3.5) with a spherical morphology and an average diameter of 10.4 µm (Figure 1o). To simulate the state of microspheres in the gastrointestinal tract and evaluate their resistance to gastric acid and responsiveness to alkaline environments, the microspheres were treated with solutions of varying pH and examined by SEM. The results showed that ZIF‐8@OXA@inulin@CAP microspheres maintained an intact spherical morphology at pH 3.0, whereas increasing pH gradually dissolved the CAP coating, thereby exposing the underlying ZIF‐8@OXA@inulin (Figure 1p). To evaluate encapsulation and release, we conducted additional studies using physiologically relevant media, including simulated gastric fluid (SGF, pH 1.2) and simulated colonic fluid (SCF, pH 6.8). As shown in Figure S2, ZIF‐8@OXA@inulin@CAP exhibited morphological stability in SGF, with SEM micrographs revealing no detectable alteration in structural integrity over the observation period. In contrast, exposure to SCF resulted in a progressive loss of the smooth spherical surface. The microspheres exhibited a roughened morphology, with ZIF‐8@OXA NPs visibly exposed at the periphery by 8 h. Building on this observation, and recognizing that inulin is metabolized by colonic microbiota, we next incubated the microspheres in SCF supplemented with *Bifidobacterium longum* (*B. longum*). Under these conditions, SEM revealed substantial erosion of the inulin matrix, with clear exposure and local aggregation of ZIF‐8@OXA NPs. The inulin matrix was nearly disintegrated at 8 h. In parallel, we quantified OXA release under the same conditions to link morphology with function. Cumulative release was minimal in SGF, accelerated in SCF, and reached its highest rate in SCF plus *B. longum*, consistent with microbiota‐assisted erosion of the inulin matrix (Figure S3). Taken together, these results substantiate true inulin‐matrix encapsulation and support a pH‐ and microbiota‐responsive, sequential release mechanism rather than superficial adsorption. Additionally, the hydrodynamic size of ZIF‐8@OXA@inulin microspheres was 6.5 µm, while the average size of ZIF‐8@OXA@inulin@CAP microspheres was 14.2 µm (Figure S4). Moreover, Figure 1q confirms the negative charge of the ZIF‐8@OXA@inulin and ZIF‐8@OXA@inulin@CAP microspheres. The ZIF‐8@OXA drug loading (DL) and encapsulation efficiency in ZIF‐8@OXA@inulin microspheres were 8.3% and 40.5%, respectively, measured using inductively coupled plasma‐mass spectrometry (ICP‐MS).

Furthermore, we characterized the ZIF‐8@OXA@Chitosan microspheres by SEM, which showed uniformly dispersible microspheres in suspension with a spheroidal morphology (Figure S5a). We further examined ZIF‐8@OXA@Chitosan@CAP microspheres by SEM under pH 3.5 conditions and observed uniformly dispersible, spherical particles with an average diameter of 11.2 µm (Figure S5b). The hydrodynamic size of ZIF‐8@OXA@ Chitosan microspheres was 6.3 µm, and ZIF‐8@OXA@Chitosan@CAP microspheres averaged 15.4 µm (Figure S5c). Zeta potential measurements confirmed that both ZIF‐8@OXA@Chitosan and ZIF‐8@OXA@Chitosan@CAP microspheres carried a negative surface charge (Figure S5d), supporting charge comparability with the systems and helping to exclude adhesion differences. Drug loading and encapsulation efficiency for ZIF‐8@OXA@Chitosan@CAP microspheres were 9.5% and 43.2%, respectively, as quantified by ICP‐MS. In addition, Supplementary Table S1 summarizes the full physicochemical comparison across ZIF‐8@OXA@inulin, ZIF‐8@OXA@inulin@CAP, ZIF‐8@OXA@Chitosan, and ZIF‐8@OXA@Chitosan@CAP microspheres. No statistically significant differences were detected among particle size, hydrodynamic size, zeta potential, drug loading, or encapsulation efficiency. Taken together, these above results collectively confirmed the appropriate dispersity and well stability of ZIF‐8@OXA@Chitosan@CAP (hereinafter termed ZIF‐8@OXA@Chitosan) and ZIF‐8@OXA@inulin@CAP microspheres (hereinafter termed ZIF‐8@OXA@inulin).

### 2.2 Cellular Internalization Effect, Intratumoral Drug Accumulation, and Pharmacokinetic Properties

**FIGURE 2:**
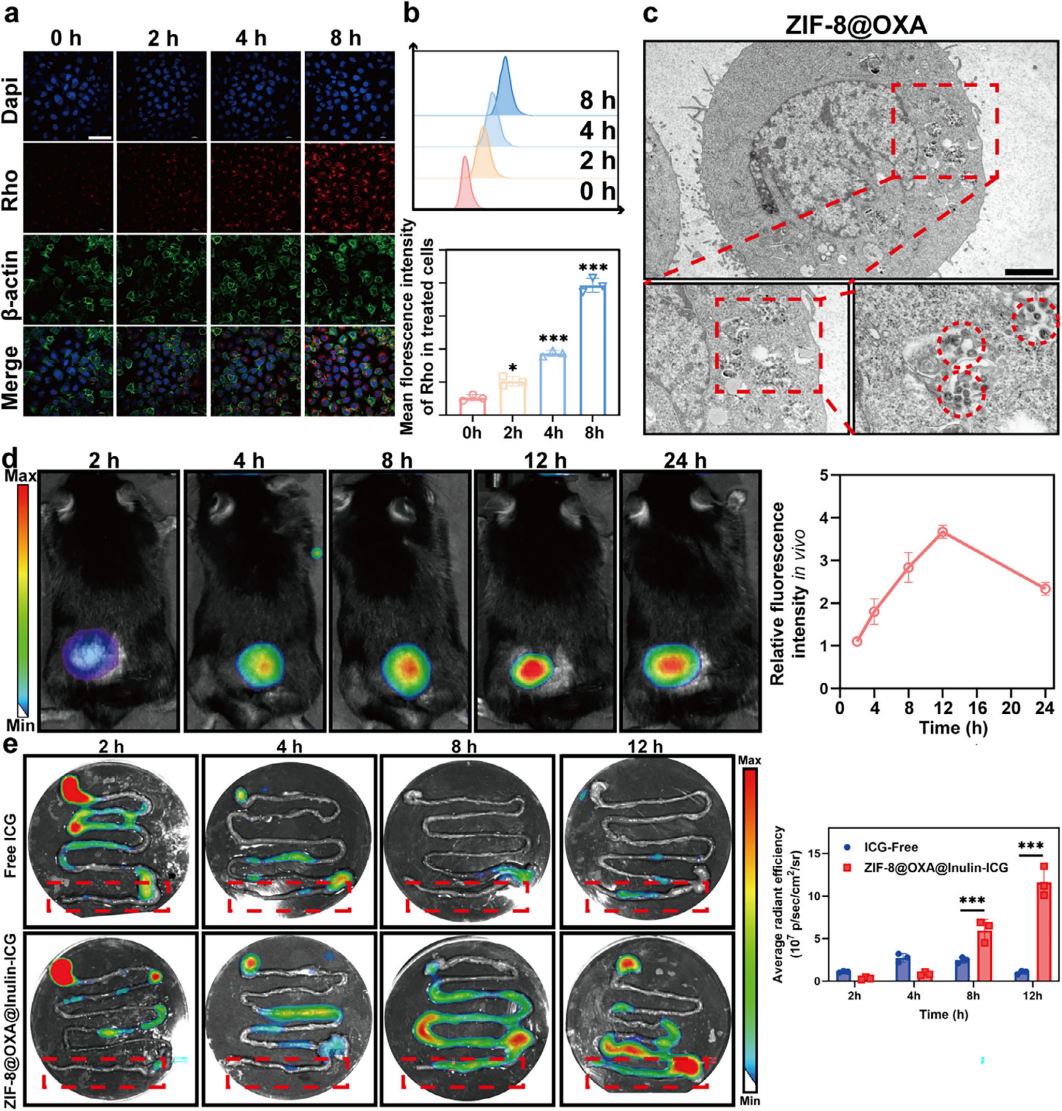
Cellular internalization effect and intratumoral accumulation of drugs. (a, b) The uptake of Rho‐labeled ZIF‐8@OXA NPs by MC38 cells over 0–8 h was observed using CLSM or FCM, with the corresponding fluorescence quantified (n = 3). Scale bar: 30 µm. (c) Bio‐TEM images demonstrating the uptake and degradation of ZIF‐8@OXA NPs by MC38 cells. Scale bar: 10 µm. (d) In vivo fluorescence images of MC38‐tumor‐bearing mice at 2, 4, 8, 12, and 24 h after treatment with Cy5.5‐labeled ZIF‐8@OXA NPs and relative fluorescence intensity. (e) Ex vivo fluorescent images and qualification of intestines from orthotopic colon tumor model mice at 2, 4, 8, and 12 h after oral administration of free ICG or ZIF‐8@OXA@inulin‐ICG (n = 3). * *p* < 0.05, ** *p* < 0.01, *** *p* < 0.001.

To determine whether ZIF‐8@OXA NPs could be internalized by MC38 cells, we labeled the NPs with rhodamine B (Rho) [29, 30]. The cellular uptake rate was assessed by measuring intracellular Rho fluorescence. Confocal laser scanning microscopy (CLSM) results indicated a gradual increase in intracellular Rho fluorescence intensity as the incubation period was extended from 0 to 8 h (Figure 2a). This suggests that the uptake rate of ZIF‐8@OXA NPs by MC38 cells was significantly time‐dependent. The flow cytometry (FCM) results demonstrated a comparable trend (Figure 2b). In addition, cell pellet specimens were observed using biological transmission electron microscopy (bio‐TEM) after incubation with ZIF‐8@OXA NPs. Figure 2c shows that ZIF‐8@OXA NPs were identifiable within the cytoplasm, and their morphology appeared less compact (**enlarged image** in Figure 2c). Moreover, a mouse model of MC38 subcutaneous tumors was established to investigate the effect of ZIF‐8@OXA NPs on drug accumulation in the tumors. Cy5.5‐labeled ZIF‐8@OXA NPs were prepared for in vivo imaging studies. As shown in Figure 2d, following intravenous (i.v.) injection, the fluorescence intensity increased over time and peaked at 12 h. Besides, from ex vivo fluorescence images of the excised organs at 12 h post‐injection, the Cy5.5‐ZIF‐8@OXA NPs preferred to accumulate in the tumor (Figure S6a,b). To clarify the biodistribution of ZIF‐8@OXA NPs, the major organs and tumor tissues were also weighted at the indicated time points after treatment, and the Zn content within these tissues was detected by ICP‐MS. Our results showed that after intravenous injection, ZIF‐8@OXA NPs was mainly accumulated in the liver and tumor (Figure S6c), indicating that ZIF‐8@OXA NPs did not interfere with the metal metabolism of the host animal. The above result indicated efficient tumor accumulation of ZIF‐8@OXA NPs consistent with enhanced permeability and retention (EPR) mediated passive targeting, thereby limiting excessive deposition in healthy organs.

To further determine the effect of ZIF‐8@OXA@inulin on drug accumulation in tumors, an orthotopic colon tumor model was established. The mice were orally administered either infrared fluorescent indocyanine green (ICG)‐loaded ZIF‐8@OXA@inulin (ZIF‐8@OXA@inulin‐ICG) or free ICG [24]. In vivo imaging using an in vivo imaging system (IVIS) revealed that both groups exhibited nearly equal signal intensities in ex vivo intestinal tissue 2 h post‐administration. However, the signal intensity in the free ICG group diminished and was nearly undetectable in mice after 8 h. In contrast, the ZIF‐8@OXA@inulin‐ICG group maintained a high signal intensity for up to 12 h, indicating that ZIF‐8@OXA@inulin‐ICG had a longer retention time in the intestine, enhanced colon‐targeting capability, and prolonged retention of ZIF‐8@OXA@inulin (Figure 2e).

To investigate the influence of inulin modification of ZIF‐8@OXA NPs on blood circulation, we performed pharmacokinetic (PK) studies in an orthotopic colon tumor model after oral or IV treatment [31]. Quantitative analysis indicated that the OXA concentrations in the liver, spleen, and kidneys of the free OXA group (IV and oral) were higher than those in the ZIF‐8@OXA@inulin group. No significant differences were observed in the intratumoral drug concentration between the OXA group (IV) and ZIF‐8@OXA@inulin group, whereas the OXA group (oral) reached a lower intratumoral concentration (Figure S7). Subsequently, the effect of dose escalation on the oral bioavailability of ZIF‐8@OXA@inulin was evaluated at doses of 5, 10, and 20 mg/kg OXA (designated ZIF‐8@OXA@inulin (5 mg/kg), ZIF‐8@OXA@inulin (10 mg/kg), and ZIF‐8@OXA@inulin (20 mg/kg), respectively). The plasma concentration‐time curves following IV and oral administration of OXA or ZIF‐8@OXA@inulin at the doses are presented in Figure S8.

### 2.3 Nuclear/Mitochondrial DNA Dual Damage and Pyroptosis Mediated by Enhancement of Zn‐Ion Overload and OXA Release

First, we conducted a cell counting kit‐8 (CCK‐8) assay to determine the anti‐tumor effect of ZIF‐8@OXA NPs. As shown in Figure S9, the CCK‐8 assay results revealed that treatment with ZIF‐8@OXA NPs led to higher cell mortality than treatment with ZIF‐8 NPs. To evaluate the therapeutic effect of ZIF‐8@OXA NPs, we performed live/dead double staining. As shown in Figure S10, ZIF‐8@OXA exhibited the highest mortality rate. Meanwhile, the proportion of 7‐AAD+ cells was the highest in the ZIF‐8@OXA group (Figure S11).

To validate biocompatibility for oral delivery systems, we performed the recommended intestinal epithelial cytotoxicity (Caco‐2/HT29‐MTX co‐culture viability assay) and immune‐cell cytotoxicity tests. Specifically, the cytotoxicity of ^F127^ZIF‐8 and ^F127^ZIF‐8@OXA NPs toward Caco‐2/HT‐29 monolayers was evaluated in a concentration‐dependent manner using both CCK‐8 viability and lactate dehydrogenase (LDH) release readouts. As shown in Figure S12a,b, after 24 h of incubation none of the tested concentrations of ^F127^ZIF‐8 and ^F127^ZIF‐8@OXA produced measurable epithelial toxicity, and the LDH assay showed a similar trend, with values comparable to vehicle controls. In parallel, we assessed immune‐cell compatibility and observed that, after 24 h exposure, the same concentration range of ^F127^ZIF‐8 and ^F127^ZIF‐8@OXA did not reduce viability in RAW264.7 macrophages, primary mouse bone‐marrow neutrophils, or CD8+ T cells (Figure S12c,d). Tight junctions in the Caco‐2/HT29‐MTX co‐culture were also confirmed by western blotting for the tight‐junction protein ZO‐1 and occludin. Western blotting result showed no significant change in ZO‐1 and occludin expression after exposure to ^F127^ZIF‐8 and ^F127^ZIF‐8@OXA, indicating that these formulations did not disrupt tight‐junction integrity or the functional status of the Caco‐2/HT29‐MTX monolayer (Figure S13c,d). Taken together with the hemolysis results, these experiments substantiate that ^F127^ZIF‐8 and ^F127^ZIF‐8@OXA NPs exhibit acceptable biocompatibility for oral delivery systems.

**FIGURE 3:**
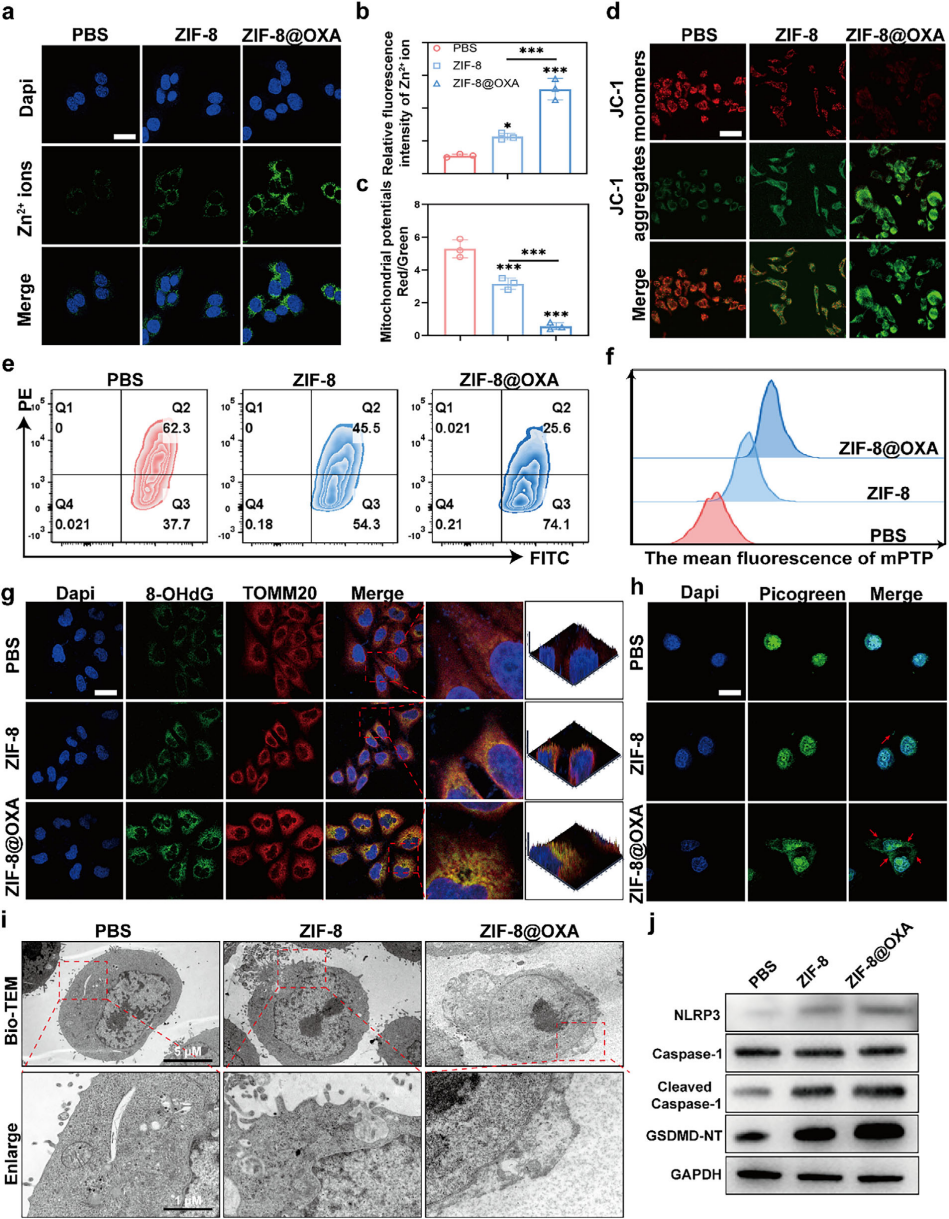
Nuclear/mitochondrial DNA dual damage and pyroptosis are evoked by the enhancement of Zn ion overload and OXA release. (a) Intracellular Zn ions detected using a Zn ion probe TSQ. Scale bar: 20 µm. (b) Statistical analysis of Zn ion levels in cells (n = 3). (c) Statistical analysis of the relative mean MMP levels in the cells (n = 3). (d) CLSM image of MMP in MC38 cells following JC‐1 probe staining. Scale bar: 40µm. (e) FCM analysis of intracellular MMP levels. (f) FCM analysis of intracellular mPTP levels. (g) MitoDNA oxidation levels. Scale bar: 20 µm. (h) DNA was detected using PicoGreen and DAPI staining. Scale bar: 20 µm. (i) Bio‐TEM of mitochondria (red boxes) in MC38 cells after various treatments. Scale bar: 5 µm (insert scale bar: 1 µm). (j) Western blotting results of NLRP3, Caspase‐1, Cleaved Caspase‐1, and GSDMD‐NT expression in MC38 cells after treatment. * *p* < 0.05, ** *p* < 0.01, *** *p* < 0.001.

To clarify the mechanism, DCFH‐DA, a biological probe, was used to detect intracellular reactive oxygen species (ROS) levels and evaluate the oxidative stress triggered by ZIF‐8@OXA NPs (Figure S14). Furthermore, previous studies have indicated that cancer cells are highly vulnerable to Zn ion overload, which may trigger pyroptosis. Intracellular Zn ions released from ZIF‐8 NPs were detected using the Zn‐ion‐sensitive probe [N‐6‐Methoxy.8‐quinolyl)‐p‐toluenesulfonamide] (TSQ) [26]. As shown in Figure 3a,b, the concentration of Zn^2+^ in MC38 cells significantly increased. The proportion of Zn^2+^ in the ZIF‐8 group was slightly lower than that in the ZIF‐8@OXA group, possibly because the latter induced greater damage via OXA release. Similar results were obtained using FCM (Figure S15). Mechanistically, the increase in intracellular Zn^2+^ after treatment with ZIF‐8 or ZIF‐8@OXA was attributable to two coupled processes. First, endocytic uptake followed by endo‐lysosomal acidification promoted ZIF‐8 dissolution and released Zn^2+^ into the cytosol. Second, OXA induced oxidative stress and mitochondrial injury, which can oxidize thiol‐rich metallothioneins, destabilize lysosomal and mitochondrial membranes, thereby amplifying the labile Zn^2+^ pool [32, 33]. Consistent with this mechanism, intracellular Zn^2+^ showed no significant difference between ZIF‐8 and ZIF‐8@OXA at 20 µg/mL, whereas at 40 and 80 µg/mL of ZIF‐8@OXA caused a significantly greater Zn^2+^ increase than that with ZIF‐8 alone (Figure S16). Pre‐exposure to OXA followed by ZIF‐8 further elevated intracellular Zn^2+^ and this amplification was attenuated by the ROS scavenger N‐acetylcysteine (NAC), indicating a ROS‐dependent mediating mechanism connecting OXA release to Zn^2+^ mobilization (Figure S17).

Emerging evidence suggests that once intracellular ROS or Zn ion levels reach the physiological threshold, they cause mitochondrial oxidative stress, leading to mitochondrial damage and dysfunction [29, 33]. Thus, the JC‐1 detection kit was used to detect the mitochondrial membrane potential (MMP) of the treated cells using FCM and CLSM to evaluate the degree of mitochondrial damage induced by ZIF‐8@OXA NPs. As shown in Figure 3c,d, the cells treated with ZIF‐8@OXA NPs showed a lower MMP than those treated with ZIF‐8 NPs and the control group. The FCM results showed a similar trend (Figure 3e). Consistent with the decrease in MMP, the opening of the mitochondrial permeability transition pore (mPTP) exhibited a similar tendency (Figure 3f; Figure S18). In addition, FCM analysis revealed that ZIF‐8@OXA NPs triggered the production of mitochondrial superoxide anions and enhanced mitochondrial ROS (mtROS) generation. The mtROS levels in cells treated with ZIF‐8@OXA NPs were significantly higher than those in cells treated with ZIF‐8 NPs and PBS (Figure S19).

Previous studies have shown that mitochondrial DNA (mtDNA) is susceptible to free radical damage owing to the absence of protective DNA‐binding proteins or histones, potentially disrupting mitochondrial biosynthesis [34, 35, 36, 37]. Consequently, we investigated whether treatment with ZIF‐8@OXA NPs led to increased mtDNA damage due to oxidative stress and elevated intracellular Zn ion levels. Next, the oxidative stress biomarker 8‐hydroxy‐2‐deoxyguanosine (8‐OHdG), which indicates oxidative mtDNA damage, was evaluated. As shown in Figure 3g, the significant colocalization of red fluorescence associated with TOMM20 and green fluorescence corresponding to 8‐OHdG suggested that most of the oxidative damage occurred within mtDNA. Moreover, OXA exerts its anticancer effects by forming platinum‐DNA adducts, leading to DNA damage [38]. We assessed the expression levels of γ‐H2AX, a well‐established marker for DNA double‐strand breaks, which is notably expressed during DNA repair processes and serves as an indicator of DNA damage. CLSM images revealed that ZIF‐8@OXA NPs increased the expression of γ‐H2AX due to OXA release and oxidative stress, indicating nuclear DNA damage (Figure S20). In addition, dsDNA was stained with PicoGreen and DAPI simultaneously. As shown in Figure 3h, PicoGreen staining revealed that the cells accumulated cytoplasmic DNA after treatment with ZIF‐8@OXA NPs, indicating the leakage of damaged nuclear DNA from the nucleus. Morphological changes in the mitochondria and cell morphology were observed using bio‐TEM. Pyroptosis induced by ZIF‐8@OXA NPs resulted in significant mitochondrial damage, characterized by noticeable swelling and cavitation (Figure 3i).

Cleavage and formation of the activated N‐terminal domain of GSDMD (GSDMD‐N) by inflammatory caspases is a recently identified pyroptosis executioner that interacts with membrane phospholipids to form pores in the cell membrane [39, 40]. Given the cell swelling observed by bio‐TEM, we determined the expression levels of critical proteins associated with pyroptosis. Figure 3j and Figure S21 demonstrate that treatment with ZIF‐8@OXA NPs resulted in markedly elevated levels of cleaved caspase‐1 (c‐cas‐1) and GSDMD‐NT, indicating the activation of a caspase‐1/GSDMD‐dependent pyroptosis pathway. Furthermore, CLSM results revealed the most intense red fluorescence in cells treated with ZIF‐8@OXA NPs (Figure S22), suggesting efficient caspase‐1 activation. In typical pyroptosis, substantial release of inflammatory molecules and cellular contents occurs, including lactate dehydrogenase (LDH) and interleukin‐1β (IL‐1β) levels. As shown in Figure S23, ZIF‐8@OXA NPs significantly enhanced LDH and IL‐1β release, with levels in the culture medium increasing compared to those in PBS and ZIF‐8 NPs. Then, we introduced MCC950 as a selective inhibitor of the NLRP3 inflammasome. In MC38 cells, ZIF‐8@OXA at a concentration of 80 µg/mL showed pronounced cytotoxicity using CCK‐8 and live/dead staining, whereas cotreatment with MCC950 at 10 µm consistently reduced cell death (Figure S24a–c). Western blotting further indicated that ZIF‐8@OXA increased c‐cas‐1 and GSDMD‐NT, consistent with activation of a c‐cas‐1 and GSDMD pathway, and this signature was markedly diminished when MCC950 was present, with decreases of NLRP3, c‐cas‐1 and GSDMD‐NT (Figure S25a). Confocal observations were consistent with these findings (Figure S25b,c). Consistent with inhibition of the upstream pathway, ELISA showed that ZIF‐8@OXA elevated extracellular LDH and IL‐1β, while coadministration of MCC950 significantly attenuated both markers (Figure S25d,e). These findings suggest that ZIF‐8@OXA induces tumor cell pyroptosis, thereby exerting antitumor effects.

### 2.4 In Vitro Immunogenic Cell Death Induction and NETs Formation through Lipid Peroxidation and Ferroptosis in Neutrophil

Chemoimmunotherapy has been recognized as a significant advancement in cancer treatments. However, only a small percentage of patients exhibited positive responses. This limited efficacy is often linked to weak antitumor immunity, which is marked by the low presence of pro‐inflammatory immune cells and a suppressive immunological environment within the TME [41, 42]. Pyroptosis, a type of programmed cell death mediated by GSDMD, is characterized by cell swelling, membrane rupture, and the release of immunostimulatory substances from cells [43, 44]. Unlike apoptosis, pyroptosis triggers a strong immune response and effectively modulates the tumor immune microenvironment, making it a promising strategy for enhancing the efficacy of chemoimmunotherapy [42].

Upon pyroptosis, tumor cells release immunogenic DMAPs and trigger immunogenic cell death (ICD), which plays a role in the activation of antitumor immune responses [45]. To confirm whether treatment with ZIF‐8@OXA NPs induced ICD activation, we first measured the release of DAMPs after various treatments. A strong translocation of CRT into the cell membrane was observed using CLSM after treating MC38 cells with ZIF‐8@OXA NPs (Figure S26a). As expected, the results of both FCM analysis and western blotting showed the same tendency (Figures S27 and S28). In addition, ZIF‐8@OXA NPs triggered HMGB1 release and ATP secretion (Figures S26b and S29). Dendritic cells (DCs) and tumor‐associated macrophages (TAMs) play essential roles in the immune response, contributing to the initiation, regulation, and maintenance of innate and adaptive immunity. A growing body of research has demonstrated that DAMPs and IL‐1β facilitate the maturation and polarization of DCs and TAMs, enhancing their capacity to process and present antigens associated with dead tumor cells. To determine whether pyroptosis can induce DC maturation and TAM polarization, bone marrow‐derived dendritic cells (BMDCs) and murine macrophages (Raw 264.7) were co‐cultured with pretreated MC38 cells, followed by staining for FCM analysis. As illustrated in Figure S30 and S31, the rates of DCs maturation and antitumor M1‐like phenotype in the ZIF‐8@OXA NPs group were higher than those in the other groups, which proved the generation of stronger immunogenicity. Furthermore, CLSM images confirmed that DC maturation and an antitumor M1‐like phenotype were activated by ZIF‐8@OXA NPs (Figures S32 and S33). To connect these pyroptosis events to immune activation rather than to a downstream byproduct, we performed a Transwell co‐culture in which MC38 cells or their supernatants were incubated with BMDCs for 24 h. FCM showed that ZIF‐8@OXA markedly increased expression of the DC maturation markers, CD80 and CD86, while inclusion of MCC950 during tumor cell treatment substantially attenuated this response, consistent with priming by soluble mediators released during pyroptosis (Figure S34).

**FIGURE 4:**
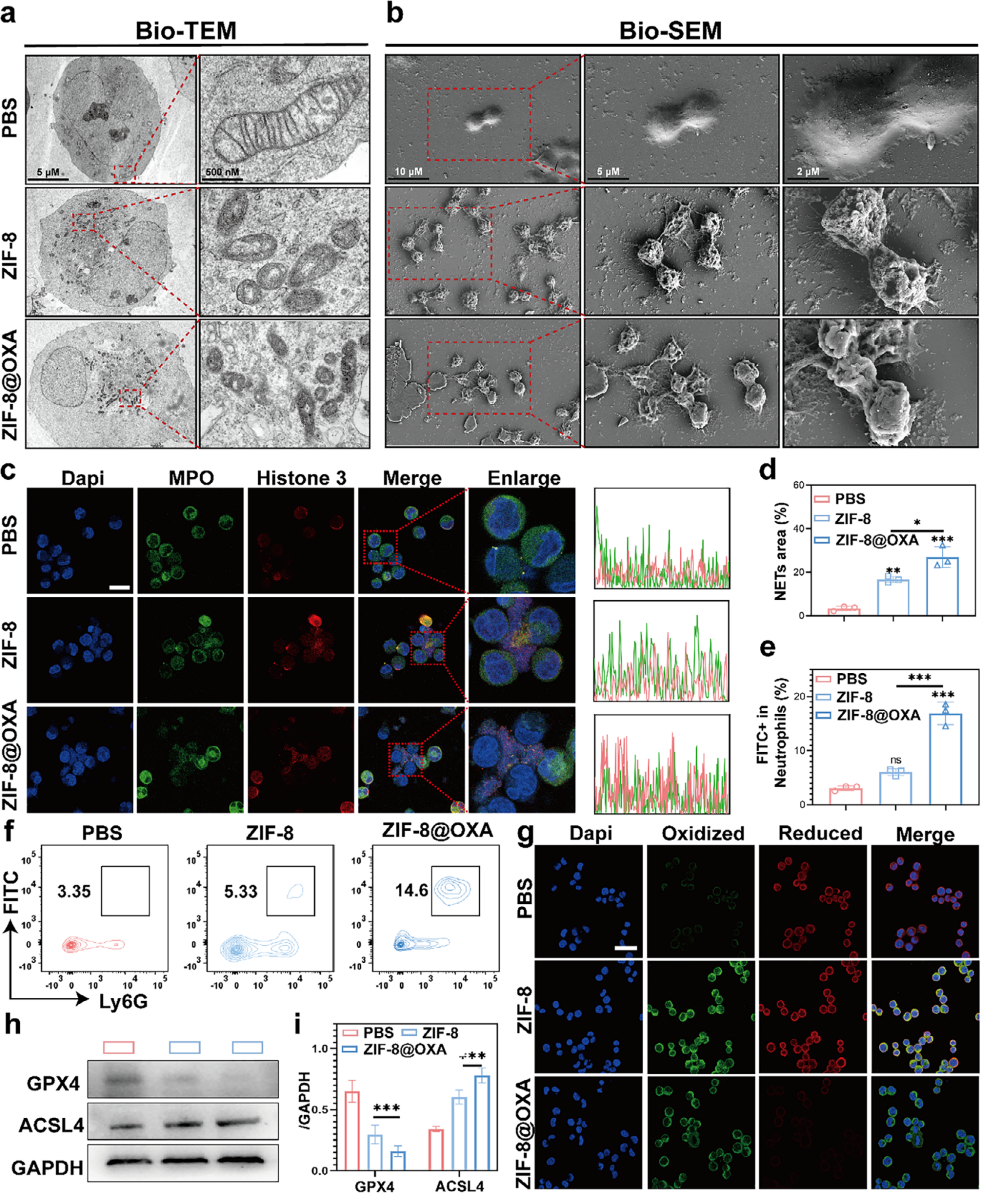
NETs formation through ferroptosis activation in neutrophils. (a) Bio‐TEM of mitochondria (red boxes) in neutrophils after various treatments, scale bar: 5 µm (insert scale bar: 500 nm). (b) Bio‐SEM of NETs (red boxes) in neutrophils after various treatments, scale bar: 10 µm (insert scale bar: 5 µm and 2 µm). (c) CLSM images of NETs (red boxes) in neutrophils after various treatments (n = 3). Scale bar: 20 µm. (d) Statistical analysis of NETs area. (e, f) Statistical and FCM analyses of LPO levels in neutrophils detected using the BODIPY581/591‐C11 probe (Oxidized, FITC) (n = 3). (g) LPO assay of neutrophils using C11‐BODIPY co‐incubation after treatment (scale bar, 20 µm). (h, i) Western blotting results and statistical analysis of GPX4, ACSL4, and GAPDH expression in neutrophils after treatment (n = 3). * *p* < 0.05, ** *p* < 0.01, *** *p* < 0.001.

Although neutrophils represent the largest proportion of leukocytes in human blood and are well known for their roles in cancer progression, their functions in chemoresistance remain poorly understood [46, 47]. Recently, NETs have attracted attention as potential therapeutic targets for non‐infectious diseases, including cancer [4, 5]. Chemotherapy does not directly affect neutrophils; instead, studies indicate that ATP is released from dying cancer cells treated with chemotherapy, driving NLRP3‐mediated IL‐1β secretion by other cancer cells [4]. Subsequently, IL‐1β promotes NET formation. Increasing evidence suggests that NET formation is induced by chemotherapy [7]. To investigate the role of neutrophils in chemotherapy, isolated neutrophils were co‐cultured with pretreated MC38 cells, and their interactions were examined using bio‐transmission electron microscopy (bio‐TEM) and bio‐scanning electron microscopy (bio‐SEM). As shown in Figure 4a, the mitochondria in the ZIF‐8@OXA NPs group exhibited fewer and shorter cristae than those in the PBS control group, suggesting a deterioration in oxidative phosphorylation, a hallmark of ferroptosis [48]. Figure 4b shows representative images of NETs evaluated using bio‐SEM analysis of the lower‐chamber medium. The ZIF‐8@OXA NPs group exhibited significant NET clusters, whereas the control group did not exhibit such clustering. The CLSM images revealed similar results (Figure 4c,d). Furthermore, SEM of the lower chamber medium from Transwell co‐cultures revealed abundant web‐like NET structures in the ZIF‐8@OXA group, whereas these structures were absent in the ZIF‐8@OXA plus MCC950 group, with neutrophils appearing as individual cells (Figure S35). Consistent with the ultrastructural observations, western blot analyses showed increased levels of NET associated proteins, including citrullinated histone H3 (H3Cit) and neutrophil elastase (NE), after ZIF‐8@OXA treatment, while co‐treatment with MCC950 reduced both markers (Figure S36). Taken together, these results indicate that NET formation is primarily attributable to pyroptosis mediated by ZIF‐8@OXA NPs

Previous studies have suggested that NETs formation is mainly due to the activation of ferroptosis in neutrophils; thus, we further investigated the expression of related molecules and proteins in neutrophils after co‐culturing with pretreated MC38 cells [48, 49, 50]. The results indicated that the levels of oxidative signals in neutrophils were elevated in the ZIF‐8@OXA NPs group (Figure 4e,f), which used a BODIPY581/591‐C11 probe to detect intracellular lipid peroxidation (LPO) levels. Meanwhile, as shown in Figure 4g, the green fluorescence was enhanced in the ZIF‐8@OXA NPs group. Furthermore, western blot analysis confirmed the downregulation of ferroptosis‐related proteins, including GPX4, and the upregulation of ACSL4 expression (Figure 4h,i). Accordingly, ferroptosis activation of neutrophils achieved by ZIF‐8@OXA NPs significantly increased the expression of the immunoinhibitory factor TGF‐β (Figure S37). Thus, ZIF‐8@OXA NPs could augment the response to chemoimmunotherapy by promoting the maturation of DCs and polarization of TAMs; however, they induced an immunosuppressive effect owing to the promotion of NETs.

### 2.5 In Vivo Therapeutic and Biocompatibility Efficacy of ZIF‐8@OXA NP‐Embedded Inulin Microspheres Against Orthotopic Colon Tumor

**FIGURE 5:**
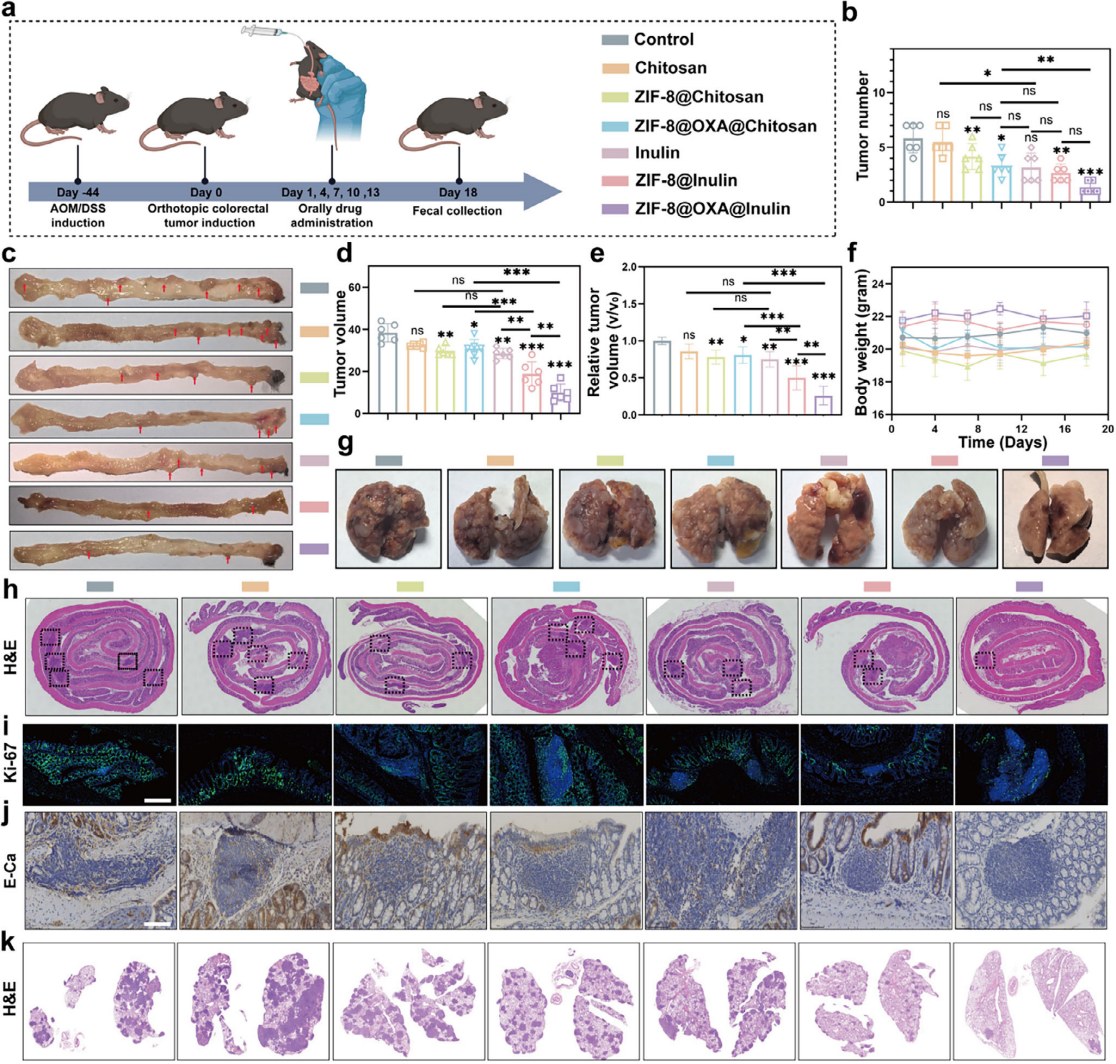
In vivo therapeutic efficacy of ZIF‐8@OXA NPs‐embedded inulin microspheres against orthotopic colon cancer. (a) Treatment schedule for ZIF‐8@OXA NP‐embedded inulin microsphere therapy for the orthotopic colon tumor model (created using BioRender.com). (b) Tumor number on day 18, when the mice were euthanized (n = 6). (c) Representative images of the colon after the different treatments. (d‐e) Tumor volume and relative tumor volume on day 18 when the mice were euthanized (n = 6). (f) Body weights of the treated mice (n = 6). (g) Photographs of the lungs after different treatments in the lung metastasis model (n = 6). (h–j) H&E, Ki67, and E‐Ca‐stained intestinal sections. (k) H&E staining images of lungs after different treatments in the lung metastasis model (n = 6). * *p* < 0.05, ** *p* < 0.01, *** *p* < 0.001.

Considering the excellent in vitro antitumor effects of ZIF‐8@OXA NPs, we constructed an orthotopic colon tumor model to evaluate the synthesized therapies in vivo. A schematic of the animal experiments is shown in Figure 5a. Mice were intraperitoneally injected with azoxymethane (AOM), followed by treatment with dextran sulfate sodium (DSS) to induce orthotopic colon tumors. Subsequently, the mice were administered ZIF‐8@OXA@inulin as scheduled. In this study, chitosan and CAP encapsulated ZIF‐8 NPs (ZIF‐8@Chitosan@CAP) or ZIF‐8@OXA NPs (ZIF‐8@OXA@Chitosan@CAP) served as control [51, 52]. For brevity, ZIF‐8@Chitosan@CAP and ZIF‐8@OXA@Chitosan@CAP are hereafter referred to as ZIF‐8@Chitosan and ZIF‐8@OXA@Chitosan, respectively. As shown in Figure 5b–e, the tumor numbers, sizes decreased remarkably in the ZIF‐8@OXA@inulin‐treated groups. Photographs of the harvested colon tissues showed similar trends (Figure 5c). A detailed analysis of the inhibitory effects of various treatments on colon tumors revealed the following ranking: Chitosan < Inulin = ZIF‐8@Chitosan = ZIF‐8@OXA@Chitosan < ZIF‐8@inulin < ZIF‐8@OXA@inulin. ZIF‐8@OXA@inulin microspheres significantly improved therapeutic outcomes, with a tumor inhibition rate of 85.3%. Furthermore, ZIF‐8@OXA@chitosan produced an anti‐tumor effect relative to that with saline, whereas coadministration of MCC950 attenuated the effect to control levels (Figure S38). Western blotting showed that ZIF‐8@OXA@chitosan increased cleaved caspase‐1 and the N‐terminal fragment of GSDMD, consistent with activation of the caspase‐1/GSDMD pathway. This signature was attenuated by the NLRP3 inhibitor, MCC950, which reduced NLRP3, cleaved caspase‐1, and GSDMD‐NT levels, indicating that the antitumor effect of ZIF‐8@OXA@chitosan is primarily attributable to pyroptosis (Figure S39). This enhanced efficacy was attributed to the therapeutic benefits of pyroptosis induced by ZIF‐8@OXA NPs, along with the synergistic effects of inulin.

Therefore, the biocompatibility of this novel oral drug delivery system is crucial. In the safety evaluation, healthy mice that received the various formulations did not exhibit significant body weight loss (Figure 5f). Furthermore, pathological analysis using hematoxylin and eosin (H&E) staining of the main organs revealed almost no significant variations, supporting the safety of the treatments (Figure S40). In addition, no notable differences were observed in the serum biochemical and hematological parameters following treatment (Figure S41). Subsequently, the intestinal sections of the treated mice were collected for H&E and immunofluorescence (IF; Ki‐67) staining. As shown in Figure 5h, the tumor areas in the H&E‐stained intestinal sections from mice treated with ZIF‐8@OXA@inulin were barely recognizable compared to those in the control group, which exhibited multiple large tumor burdens. In addition, cell proliferation was significantly inhibited in the ZIF‐8@OXA@inulin group, as evidenced by Ki‐67 staining results (Figure 5i).

Several studies have demonstrated that chemotherapy‐induced NETs promote metastasis by directly interacting with cancer cells or creating an immunosuppressive environment at the primary or distant tumor site [4, 5, 7]. We then compared the inhibitory effects of the different groups on pulmonary tumor metastasis. As shown in Figure 5g, pulmonary tumor metastasis appeared in the control mice, and the formation of tumor nodules was suppressed in the ZIF‐8@OXA@inulin group. In addition, lower expression of E‐cadherin (E‐Ca) was observed in the intestinal sections of mice with pulmonary tumor metastasis (Figure 5j). Furthermore, significantly fewer metastatic nodules were observed in the lungs of the ZIF‐8@OXA@inulin group, as evidenced by H&E staining (Figure 5k; Figure S42), indicating that the synergistic effects of inulin drastically decreased the systemic side effects of chemotherapy, including metastasis. Taken together, these results consistently demonstrate that ZIF‐8@OXA@inulin exhibits enhanced tumor cell‐killing effects and antitumor metastasis in vivo.

### 2.6 In Vivo Antitumor Immune Response through Inhibition of NET Formation by ZIF‐8@OXA NP‐Embedded Inulin Microspheres

**FIGURE 6:**
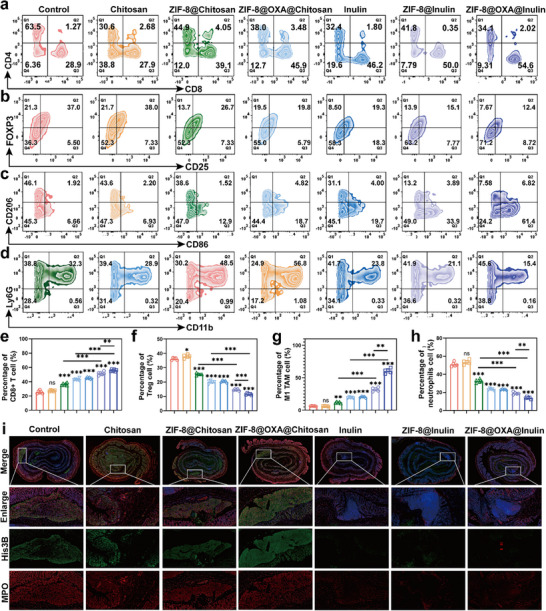
In vivo antitumor immune response through inhibition of NET formation by ZIF‐8@OXA NPs‐embedded inulin microspheres. (a) Analysis of CD8+ T cells (CD45+CD3+CD8+) in colon samples extracted from mice subjected to the indicated treatments using FCM (n = 6). (b) Representative FCM analysis of Tregs (CD45+CD3+ CD4+Foxp3+CD25+) infiltrating the orthotopic colon cancer after receiving different treatments (n = 6). (c) Representative FCM analysis of M1‐like macrophages (CD45+CD11b+F4/80+CD80+) and M2‐like macrophages (CD45+CD11b+F4/80+ CD206+) (n = 6). (d) Representative FCM analysis of neutrophils (CD45+CD11b+Ly6G+) infiltrating the orthotopic colon cancer after different treatments (n = 6). (e–h) Statistical evaluation of the infiltration of CD8+ T‐cells, Tregs, TAMs, and neutrophils in orthotopic colon tumors (n = 6). (i) Representative immunostaining of neutrophils (red, MPO), NETs (green, His3B), and DNA (blue, DAPI) in the colon tissues of mice treated as indicated. * *p* < 0.05, ** *p* < 0.01, *** *p* < 0.001.

To investigate the synergistic effects of dual damage to nuclear/mitochondrial DNA mediated by pyroptosis and the impact of inulin on the activation of antitumor immunity, we collected immune cells derived from tumor tissues and tumor‐draining lymph nodes (TDLNs) for comparative analysis. In this study, we determined the maturation rate of DCs within TDLNs to evaluate the activation of antitumor immune responses. As shown in Figures S43 and S44a, the ZIF‐8@OXA@Chitosan group exhibited a slight increase in the maturation rate of DCs (approximately 13.6%) compared to the saline group. This increase is primarily attributed to the dual damage to nuclear and mitochondrial DNA induced by pyroptosis and the subsequent effect of ICD. In contrast, the proportion of mature DCs was significantly increased in the ZIF‐8@OXA@inulin group (∼29.0%), suggesting that this two‐pronged approach includes ICD induction and inulin regulation. In addition, the average percentage of cytotoxic T cells (CD3+CD8+) within the tumor tissues treated with ZIF‐8@OXA@inulin was 54.6%, which was remarkably higher than that in the ZIF‐8@OXA@Chitosan (45.9%), ZIF‐8@Chitosan (39.1%), and saline groups (28.9%) (Figure 6a,e; Figure S44b). Moreover, it is worth noting that immunosuppressive regulatory T cells (Tregs) tend to proliferate and adapt within an immunosuppressive microenvironment; consequently, immunomodulation using inulin in conjunction with pyroptosis‑inducing NPs may limit Treg expansion and enhance antitumor immune responses. As shown in Figure 6b,f and Figure S44c, the percentage of Tregs in the ZIF‐8@OXA@inulin group was effectively suppressed. Subsequently, the TAM phenotypes within the tumor tissues were further characterized using FCM to examine the antitumor immune response. As expected, our results showed that the ZIF‐8@OXA@inulin group exhibited the lowest proportion of M2‐type TAMs (CD11b+F4/80+CD206+) and a remarkable increase in the number of M1‐type TAMs (CD11b+F4/80+CD80+) by approximately 10‐fold compared to the saline group (Figure 6c,g; Figures S45 and S44d). Neutrophils, the most prevalent type of leukocyte in peripheral blood, are recognized as the first line of immune defense against infections and inflammatory responses. Recent studies have provided increasing evidence that neutrophil depletion or NETs can significantly diminish the immune response [4, 5, 7, 48]. We then assessed the proportion of neutrophils present in the tumors following the various treatments. The reduced neutrophil infiltration into tumors further supports the notion that inulin can reprogram the immunosuppressive TME, as neutrophils have the potential to impede antitumor immune responses. As illustrated in Figure 6d,h and Figure S44e, the frequency of neutrophils (CD11b+Ly6G+) in the ZIF‐8@OXA@inulin group was the lowest among all groups, whereas an increase in neutrophil frequency was noted in the ZIF‐8@Chitosan‐ and ZIF‐8@OXA@Chitosan‐treated mice, which were 2.29‐fold and 3.68‐fold higher than those in the ZIF‐8@inulin and ZIF‐8@OXA@inulin groups, respectively.

Next, we extended the analysis in vivo to determine whether pyroptosis induced by ZIF‐8@OXA drives infiltration of DCs and neutrophils. As shown in Figure S46, treatment with the ZIF‐8@OXA@chitosan increased the maturation rate to approximately 10.1% compared with that of saline controls, whereas coadministration of MCC950 reduced it to approximately 6.87%. We further quantified intratumoral neutrophils and the ZIF‐8@OXA@chitosan group exhibited the highest frequency at approximately 19.4%, which decreased to approximately 7.38% when MCC950 was co‐administered (Figure S47). These coordinated changes in DC maturation and intratumoral neutrophil infiltration, observed under ZIF‐8@OXA treatment and reversed by inhibition of the NLRP3 pathway, support a causal role for pyroptosis as an upstream driver of DC maturation and neutrophil recruitment. Taken together, the data indicate that ZIF‐8@OXA exerts antitumor activity primarily through induction of pyroptosis, which acts upstream to promote DC maturation in tumor‐draining lymph nodes and neutrophil infiltration within tumors. Furthermore, western blotting revealed elevated citrullinated histone H3 and neutrophil elastase after ZIF‐8@OXA treatment, both of which were reduced by MCC950, indicating that pyroptosis induced by ZIF‐8@OXA@chitosan causes the adverse effect of NETs formation (Figure S44). IF images of the tumor tissues also showed that ZIF‐8@Chitosan and ZIF‐8@OXA@Chitosan promoted NETs formation, whereas ZIF‐8@inulin and ZIF‐8@OXA@inulin inhibited NETs formation, revealing that inulin modification could inhibit neutrophil aggregation and, possibly, the subsequent release of NETs (Figure 6i; Figure S49). In this context, ZIF‐8@OXA NPs elicited an antitumor immune response by inducing pyroptosis, which subsequently triggered ICD. In addition, inulin modification enhanced immune regulation by inhibiting the formation of NETs.

### 2.7 Gut Microbiota Regulation Effects

The gut microbiome has garnered significant attention because of its crucial impact on human physiology and biological processes [16, 18]. Emerging evidence indicates that microbial dysbiosis frequently occurs during chemotherapy [13, 15]. We investigated whether treatment with ZIF‐8@OXA NPs embedded in an inulin microsphere (ZIF‐8@OXA@inulin) could prevent chemotherapy‐induced dysbiosis. Mice were divided into groups and administered chemotherapeutic agents (ZIF‐8@OXA NPs), either with or without embedded inulin, while maintaining a consistent dosage and timing. Colon content samples were collected, and 16S ribosomal RNA gene sequencing was performed in the V3V4 region. A schematic representation of the animal experiments is presented in Figure S50.

**FIGURE 7:**
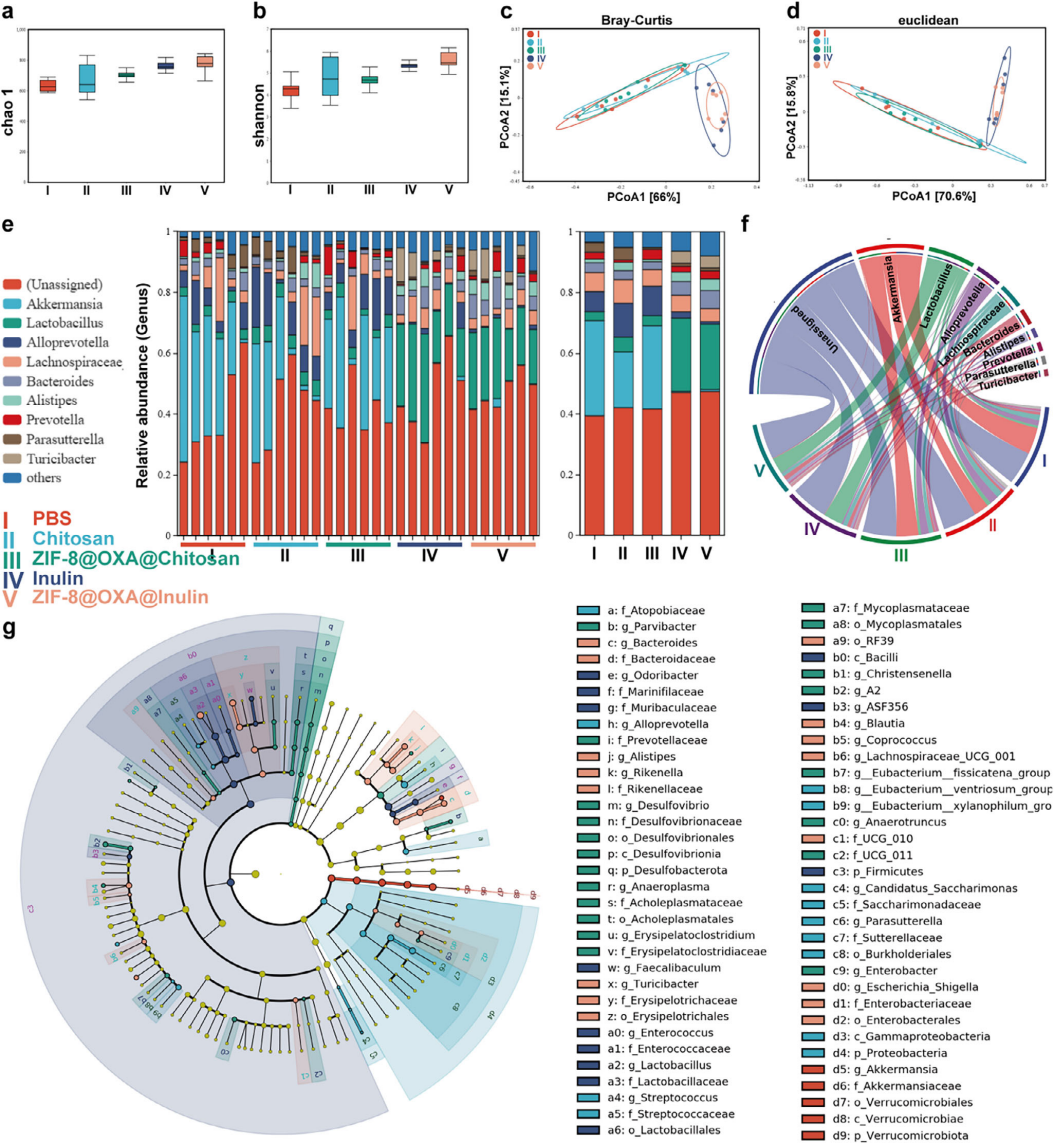
Effects of gut microbiota regulation. (a, b) Microbial α‐diversity in terms of Chao 1 and Shannon indices (n = 6). (c, d) Microbial β‐diversity PCoA analysis based on Bray–Curtis and Euclidean distances (n = 6). (e) Relative abundance of gut microbiota at the genus level. (f) Chord diagram revealing the top 10 most abundant phyla of the bacterial communities in the five groups. (g) LefSe analysis cladogram representing the significantly different taxa between groups from the Kingdom to OTU levels (LDA > 2, *p <* 0.05).

Chemotherapy without inulin (ZIF‐8@OXA@Chitosan) resulted in significant microbial dysbiosis in mice, marked by a sharp decrease in taxonomic diversity. Analysis of the colon contents showed that inulin and ZIF‐8@OXA@inulin treatment significantly improved bacterial richness (observed operational taxonomic units, OTUs) and maintained alpha diversity (Shannon and Chao1 diversity indices) compared with the chemotherapy‐only group (Figure 7a,b). Visualization of beta diversity using Bray–Curtis and Euclidean distances obtained from principal coordinate analysis (PCoA) and non‐metric multidimensional scaling (NMDS) plots (Figure 7c,d; Figure S51) demonstrated that the inulin plus chemotherapy group (ZIF‐8@OXA@inulin) exhibited a distinct gut flora profile compared to the chemotherapy‐alone group (ZIF‐8@OXA@Chitosan). At the genus level, the ZIF‐8@OXA@inulin and inulin groups exhibited a significant increase in the abundance of beneficial genera, such as *Alistipes*, *Lactobacillus*, *Enterorhabdus*, and *Turicibacter*, along with a decrease in harmful taxa, such as *Alloprevotella* and *Parasutterella*, which can inhibit the formation of NETs [12], modulate the TME, and enhance the immune response, thereby promoting the efficacy of chemoimmunotherapy [4]. Furthermore, an increase in inulin‐digesting *Bacteroides* was observed following inulin and ZIF‐8@OXA@inulin treatment (Figure 7e,f; Figure S52). The results were further validated using linear discriminant analysis effect size (LEfSe) (Figure 7g; Figure S53). Our study showed that inulin and NP‐embedded inulin could modulate the structure of intestinal flora communities and effectively alter the composition of the gut microbiota, thereby enhancing the effects of cancer chemoimmunotherapy.

### 2.8 Potent Antitumor and Immune Response Efficacy of ZIF‐8@OXA NP‐Embedded Inulin Microspheres plus αPD‐1 Therapy through Gut Microbiota Regulation

**FIGURE 8:**
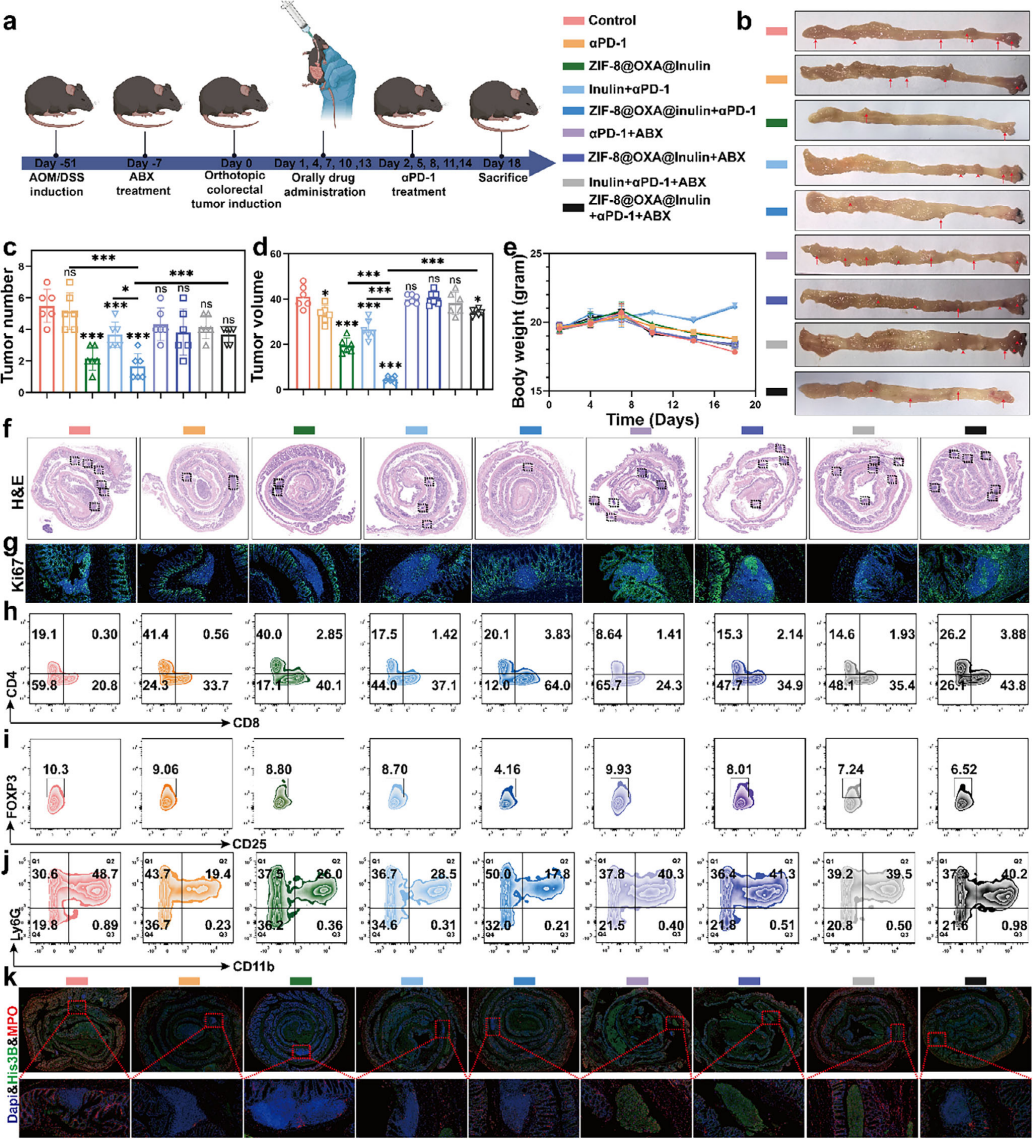
Potent antitumor and immune response efficacy of ZIF‐8@OXA NPs‐embedded inulin microspheres plus αPD‐1 therapy through gut microbiota regulation. (a) Treatment schedule (created using BioRender.com). (b) Representative images of the colon after different treatments. (c–e) Tumor number, tumor volume, and body weight of the treated mice (n = 6). (f, g) H&E‐ and Ki67‐stained intestinal sections. (h) Analysis of CD8+ T cells (CD45+CD3+CD8+). (i) Representative FCM analysis of Tregs (CD45+CD3+CD4+ Foxp3+CD25+). (j) Representative FCM analysis of neutrophils (CD45+CD11b+Ly6c+). (k) Representative immunostaining of neutrophils (red, MPO), NETs (green, His3B), and DNA (blue, DAPI) in intestinal sections. * *p* < 0.05, ** *p* < 0.01, *** *p* < 0.001.

Chemoimmunotherapy is considered one of the most promising strategies for treating malignant tumors; however, its therapeutic efficacy in advanced CRC is limited [3]. Fortunately, our in vitro and in vivo experiments showed that ZIF‐8@OXA@inulin can reverse the immunosuppressive TME by inducing pyroptosis, inhibiting NET formation, and regulating the gut microbiota. ZIF‐8@OXA@inulin may also have a significant positive effect on augmenting the therapeutic effect of immune checkpoint blockade (ICB) therapy. To verify this, the combined antitumor efficiency of ZIF‐8@OXA NP‐embedded inulin microspheres (ZIF‐8@OXA@Inulin) and αPD‐1 was further evaluated in an orthotopic colon tumor model. In addition, an antibiotic cocktail (ABX) was administered to suppress the gut microbiota, further validating that inulin enhances chemoimmunotherapy by modulating the gut microbiota (Figure 8a). As depicted in Figure 8b–d, photographs of the isolated colon tissue from different treatment groups further illustrated that the combination of ZIF‐8@OXA@inulin with αPD‐1 monotherapy resulted in the most effective treatment. The tumor suppression rate was only 18.6% with αPD‐1 treatment alone. In contrast, the combination of inulin or ZIF‐8@OXA@inulin with αPD‐1 treatment resulted in significantly increased tumor suppression rates of 39.3% and 89.5%, respectively. However, the tumor suppression rates were only 16.7% in the ZIF‐8@OXA@Inulin plus αPD‐1 with ABX groups, indicating that gut microbiota regulation significantly influenced the efficacy of cancer chemoimmunotherapy. Biocompatibility was evidenced by the negligible changes in body weight (Figure 8e). Moreover, the largest damaged area was observed in the ZIF‐8@OXA@inulin with αPD‐1 group, as evidenced by H&E staining (Figure 8f). In addition, as shown by the Ki‐67 staining results, cell proliferation was greatest inhibited in the ZIF‐8@OXA@inulin microspheres with αPD‐1 (Figure 8g).

Subsequently, tumor tissues were collected to obtain a single‐cell suspension, and the percentages of CD8+ T cells and Tregs were determined using FCM. Compared with ZIF‐8@OXA@inulin alone, ZIF‐8@OXA@inulin combined with αPD‐1 elicited a significant increase in intratumoral CD8+ T‐cell infiltration, indicating that this formulation can be paired with checkpoint blockade to further enhance anti‐tumor efficacy (Figure 8h; Figure S54a). In addition to enumerating total CD8+ T cells, we assessed functional activation of cytotoxic CD8+ T cells by intracellular cytokine staining for interferon‐γ (IFN‐γ) followed by FCM. Compared with the ZIF‐8@OXA@inulin group, the ZIF‐8@OXA@inulin with anti‐PD‐1 (αPD‐1) group produced a significant increase in the frequency of intratumoral IFN‐γ+CD8+ T cells, indicating enhanced effector activation and supporting the rationale that our formulation can be paired with checkpoint blockade to further improve antitumor efficacy. To examine microbiota dependence, we included an antibiotic cocktail (ABX) cohort. ABX administration attenuated the proportion of IFN‐γ+CD8+ T cells observed in the ZIF‐8@OXA@inulin plus αPD‐1 group, which is consistent with a key role for inulin‐mediated modulation of the gut microbiota in augmenting the immune response to checkpoint therapy (Figure S55). In addition, the percentage of Tregs in the ZIF‐8@OXA@inulin with αPD‐1 group was significantly decreased to 4.16%, whereas it decreased to 9.06% in the αPD‐1 monotherapy group (Figure 8i; Figure S54b). Moreover, the neutrophil (CD11b+Ly6G+) frequency in the ZIF‐8@OXA@inulin and αPD‐1 groups was the lowest among all groups. In contrast, an increase in neutrophil frequency was observed in the control and antibiotic (ABX)‐treated groups (Figure 8j; Figure S54c). The potent antitumor and immune response efficacies of ZIF‐8@OXA@inulin were reversed by ABX treatment, revealing that gut microbiota regulation plays an important role in chemoimmunotherapy. IF images of tumor tissues also showed that ZIF‐8@OXA@inulin with ABX promoted NETs formation, whereas ZIF‐8@OXA@inulin inhibited it, revealing that inulin modification could inhibit neutrophil aggregation due to gut microbiota regulation (Figure 8k). Taken together, the combination of ZIF‐8@OXA@inulin with αPD‐1 has the potential to elicit a robust antitumor immune response in CRC treatment through the gut microbiota regulation.

## 3 Discussion

In our study, we prepared ZIF‐8@OXA NPs that showed uniform hydrodynamic size and good aqueous dispersibility. We then developed an orally administered ZIF‐8@OXA@inulin. SEM revealed NPs densely distributed on the microsphere surface, confirming successful fabrication of ZIF‐8@OXA@inulin. Given the complexity of the GI milieu, including gastric acid, digestive enzymes, and intestinal fluids, we further applied a CAP coating to obtain a CAP coated variant of ZIF‐8@OXA@inulin (ZIF‐8@OXA@inulin@CAP, referred to as ZIF‐8@OXA@inulin). This outer CAP layer enhanced stability under GI conditions. In vitro, the particles remained intact in simulated gastric fluid, whereas in simulated intestinal fluid the CAP gradually eroded. Additionally, the inulin core underwent bacteriolytic degradation by *B. longum*, leveraging the inherent fermentability of inulin [53]. As a result, this system provides controlled release throughout the GI tract while also delivering the prebiotic inulin to enable potential drug and prebiotic synergy. The formulation exhibited favorable tolerability even at relatively high doses, consistent with its food grade composition and oral administration [23, 54, 55, 56].

Notably, ZIF‐8@OXA@inulin microspheres exhibit colon targeting with prolonged intraluminal retention. Pharmacokinetic profiling indicated that ZIF‐8@OXA NPs released in the intestine may undergo the canonical adhesion‐uptake‐transport cascade across small intestinal villi, with levels in the liver exceeding those in other organs at several time points. Importantly, this did not raise biosafety concerns. In most tissues, the NPs remain at low levels and largely encapsulated until colonic release, where bacterial digestion of inulin triggers drug liberation. These findings are in agreement with the in vivo biocompatibility assessment, including H&E histology of major organs, serum chemistry parameters and complete blood counts. In an orthotopic colon tumor model, oral ZIF‐8@OXA@inulin microspheres yielded intratumoral oxaliplatin concentrations that were not significantly different from those observed with intravenous OXA, consistent with EPR‐mediated tumor entry after colonic release [57]. Taken together, these findings substantiate controlled release within the GI tract, a favorable in vivo safety profile, and potential for clinical translation.

Mechanistically, we first demonstrated in vitro that ZIF‐8@OXA NPs induce Zn^2+^ overload together with OXA release, resulting in dual nuclear and mitochondrial DNA damage and, consequently, pyroptosis [26, 33, 40]. This response was attenuated by the NLRP3 inhibitor, MCC950 [58], supporting pathway specificity. While the same stimulus enhanced DC maturation and macrophage polarization, it concomitantly elicited an undesirable increase in NET formation, an effect attenuated by MCC950. NETs, composed of decondensed chromatin and histone scaffolds, extruded by activated neutrophils, are implicated in tumor recurrence, metastatic dissemination, and therapeutic resistance [4, 5, 21, 59]. Accordingly, interventions that curtail NET formation, including modulation of the gut microbiota, are critical for optimizing the efficacy of chemoimmunotherapy. Inulin, an FDA approved oral excipient, protects payloads against acidic and enzymatic degradation in the GI tract and can remodel the microbiota to potentiate antitumor immunity [21]. Although ZIF‐8@OXA@chitosan microspheres achieved measurable antineoplastic activity in vivo, attributable to pyroptosis‐mediated tumor cell death, their therapeutic performance remained substantially lower than that of ZIF‐8@OXA@inulin microspheres. Moreover, the chitosan formulation facilitated intratumoral NET accumulation in the colon, compromising antitumor immunity, while ZIF‐8@OXA@inulin microspheres promoted NET accumulation and potentiated antitumor immune responses. This conclusion was supported by polarization toward an M1‐like macrophage phenotype, maturation of DCs, increased intratumoral CD8+ T cell infiltration with functional activation, and decreased Treg frequencies, collectively highlighting the immunomodulatory synergy of the inulin platform in CRC.

In light of the central involvement of intestinal microbial homeostasis in tumor progression, we characterized alterations in intestinal microbiota composition. ZIF‐8@OXA@inulin and inulin alone similarly enriched beneficial genera, such as *Alistipes*, *Lactobacillus*, *Enterorhabdus*, and *Turicibacter*, and reduced taxa linked to adverse inflammation, such as *Alloprevotella* and *Parasutterella*, changes that are consistent with attenuated NETs formation. Such community level alterations, rather than species specific effects, are consistent with earlier findings on oral inulin preparations and are likely to underpin the observed immunologic reprogramming. Therefore, the combination of ZIF‐8@OXA@inulin with αPD‐1 therapy achieved robust tumor control. This conclusion was supported by a reduction in NET formation, increased activation of CD8+T cells with expansion of IFNγ+CD8+ effector cells, and further depletion of intratumoral Tregs. Antibiotic treatment used to deplete the gut microbiota (ABX) attenuated these effects, indicating that inulin‐mediated microbial modulation contributes to suppressing NET formation and potentiating antitumor immunity. Taken together, these findings provide a therapeutic rationale for integrating prebiotics with cytotoxic agents and checkpoint based immunotherapies in CRC.

In summary, we developed an orally administered inulin based microsphere system encapsulating ZIF‐8‐based NPs loaded with OXA, referred to as ZIF‐8@OXA@inulin microspheres. We show that this platform synergizes with αPD‐1 therapy and elicits potent CD8+ T cell responses while reshaping the commensal microbiota to further augment immune checkpoint blockade. Mechanistically, ZIF‐8@OXA@inulin enhances chemoimmunotherapy by triggering tumor cell pyroptosis, restraining trap formation, and fostering colonization by beneficial taxa. Notably, previous studies reported that a monotonous high‐dose free inulin diet for 14 days before inoculation slowed tumor growth without synergizing with αPD‐1. In contrast, our colon retentive, intermittently dosed ZIF‐8@OXA@inulin regimen markedly amplified αPD‐1 efficacy in a therapeutic setting. Beyond fecal microbiota transplantation and defined probiotic consortia, this dietary fiber based, orally delivered materials strategy offers a practical and safe avenue to potentiate immune checkpoint therapies. Engineering biomaterials to manipulate the gut microbiota in situ provides a generalizable framework for safely tuning systemic immunity, an approach with implications across disease areas beyond oncology.

## 4 Conclusion

We developed a CRC therapeutic strategy that integrates intestinal environmental regulation. The inulin derivative‐based ZIF‐8@OXA@inulin microspheres represent a facile and biocompatible CRC‐targeting oral drug delivery paradigm that achieves controlled release in the GI tract, enhanced intratumoral drug concentration, TME reprogramming, and gut microbiota regulation. Furthermore, ZIF‐8@OXA@inulin has potential as an adjuvant to first‐line chemotherapy. It also efficiently controlled tumor progression when combined with the immunotherapeutic agent αPD‐1 in an orthotopic colon tumor model. Thus, ZIF‐8@OXA@inulin is of practical significance and promising for CRC treatment.

## Funding

This work was supported by grants from the National Natural Science Foundation of China (Nos. 82272062 and 82372118) and the Natural Science Foundation of Guangdong Province (No. 2024A1515010390).

## Ethics Statement

All animal procedures were approved by Nanfang Hospital Experimental Animal Ethics Committee (IACUC‐LAC‐20231219‐002), and all investigation procedures were carried out in accordance with the Helsinki Declaration.

## Conflicts of Interest

The authors declare no conflicts of interest.

## Supporting information




**Supporting File**: advs74154‐sup‐0001‐SuppMat.docx.

## Data Availability

The data that support the findings of this study are available on request from the corresponding author. The data are not publicly available due to privacy or ethical restrictions.
