# Supplementary material for: Inulin‐Based Oral Chemotherapy Modulates Gut Microbiota and Immune Microenvironment through Inhibition of Neutrophil Extracellular Trap Formation for Improving Cancer Therapy
Source: Adv Sci (Weinh). 2026 Jan 29;13(20):e14318. doi: 10.1002/advs.202514318 (PMC13067827; doi:10.1002/advs.202514318)
Supplement: Supplementary file 1 — Supporting File: advs74154‐sup‐0001‐SuppMat.docx. [file ADVS-13-e14318-s001.docx]

Supporting Information

**Inulin-based Oral Chemotherapy Modulates Gut Microbiota and Immune Microenvironment through Inhibition of Neutrophil Extracellular Trap Formation for Improving Cancer Therapy**

Zhenhao Li, Jiaqiang Jiang, Yuping Zhou, Xinyuan Mao, Haotian Wen, Zhian Chen, Yutong Wang, Cong Huang, Yanfeng Hu

Z. Li, J. Jiang, X. Mao, H. Wen, Z. Chen, Y. Wang, Y. Hu

Department of General Surgery, Guangdong Provincial Key Laboratory of Precision Medicine for Gastrointestinal Tumors, Nanfang Hospital, Southern Medical University, Guangzhou 510515, China

E-mail: Y. Wang, [956940330@qq.com](mailto:956940330@qq.com); Y. Hu, [banby@smu.edu.cn](mailto:banby@smu.edu.cn)

Y. Zhou

South China University of Technology School of medicine

C. Huang

The First Affiliated Hospital of Shantou University Medical College, Shantou 515041, China
E-mail: [chuang200628@163.com](mailto:chuang200628@163.com)

1.Materials and Methods

1.1 Materials

Indocyanine Green (ICG), 2-methylimidazole (99%) and Pluronic F127 (F127) were purchased from Sigma-Aldrich. Zinc nitrate hexahydrate (Zn(NO_3_)_2_•6H_2_O) was purchased from Xilong Scientific. Cellulose acetate phthalate (CAP) and Inulin from chicory was purchased from Sigma-Aldrich. Oxaliplatin was purchased from Sanofi-Aventis.

Calcein/PI Cell Viability/Cytotoxicity Assay Kit, apoptosis detection reagent kit, CCK-8 detection Kit, 2-(4-Amidinophenyl)-6-indolecarbamidine dihydrochloride (DAPI), and mitochondrial membrane potential assay kit (JC-1) were obtained from Beyotime Inst. Biotech (Shanghai, China). Mitochondrial membrane permeability transition pore (mPTP) detection kit was purchased from Shanghai Yisheng Biotechnology Co. (Shanghai, China). N-(6-Methoxy-8-quinolyl)- p-toluenesulfonamide] (TSQ) was obtained from AAT Bioquest Inc. (Sunnyvale, USA). Annexin V-FITC/7-AAD apoptosis kit was purchased from Solarbio. Anti-8-OHdG, Anti-NLRP3, anti-Caspase-1, anti-Cleaved caspase-1, anti-GSDMD, anti-FSP1, anti-ACSL4 and anti-GPX4 antibody were obtained from Cell Signaling Technology (MA, USA). Anti-γ-H2AX (1:1,000) was purchased from Millipore. BODIPY581/591-C11 probe and PicoGreen® was obtained from Thermo Fisher Scientific (Grand Island, NY). Mouse IL-1β and tumor necrosis factor-β (TNF-β) ELISA kits were purchased from eBioscience. Lactic acid dehydrogenase (LDH) assay kit was from Jiancheng Biotech (Nanjing, China). Anti-CRT, anti-HMGB1 anti-Histone H3, anti-myeloperoxidase antibodies were purchased from Abcam Co. (Cambridge, UK). Anti-CD45, anti-CD3, anti-CD4, anti-CD8, anti-Foxp3, anti-CD25, anti-CD11b, anti-F4/80, anti-CD206 and anti-Ly6G were purchased from BD Biosciences. The PBS, Dulbecco’s modified Eagle’s medium (DMEM), and fetal bovine serum (FBS) were obtained from Gibco Laboratories (NY, USA). Stain Buffer and Fixable Viability Stain were purchased from BD Pharmingen (NJ, USA). Murine colon adenocarcinoma cells (MC38) and *Bifidobacterium longum* (BL) were purchased from American Type Culture Collection (ATCC). All chemical agents were of analytical grade and were used directly with no further purification.

1.2 Synthesis of Zeolitic imidazolate framework-8 (ZIF-8) nanoparticles (NPs)

11.3 mL of methanol containing 297.5 mg of Zn(NO_3_)_2_•6H_2_O was added into 11.3 mL of 2-methylimidazole (660 mg) methanol solution. The mixed solution was further stired at room temperature for 60 min. The products were centrifuged (10000 rpm for 8 min), washed, and then redispersed in 10 mL of water for further usage.

1.3 Synthesis of ZIF-8@OXA NPs

11.3 mL of methanol containing 297.5 mg of Zn(NO_3_)_2_ •6H_2_O and 20 mg of oxaliplatin (OXA) was added into 11.3 mL of 2-methylimidazole (660 mg) methanol solution. The mixed solution was further stired at room temperature for 60 min. The products were centrifuged (10000 rpm for 8 min), washed, and then redispersed in 10 mL of water for further usage. 10 mL of ZIF-8 NPs or ZIF-8@OXA NPs aqueous solution was added into 20 mL of deionized water containing 100 mg of F127. The mixed solution was further stired at room temperature for 12 h. The products were centrifuged (10000 rpm for 8 min) and washed with deionized water for further usage.

1.4 Synthesis of ZIF-8@OXA@inulin microspheres

To coat the surface of the ZIF-8@OXA NPs with inulin, 1 mg of ZIF-8@OXA NPs previously prepared were dispersed in 1 mL of distilled water and sonicated in an ultrasonic bath for 10 min. Simultaneously, a dispersion of inulin was prepared at 10% (w/v) in distilled water and stirring for 3 h at the room temperature. Then, 1 mL ZIF-8 solution and 100 mL inulin solution were mixed at room temperature and stirred at 500 rpm for 24 h. Finally, to remove the excess of inulin, the material was precipitated by centrifugation at 1500 g for 10 min, washed once with distilled water and stored in 1 mL distilled water. As a control, ZIF-8@OXA@Chitosan microspheres was synthesized by replacing inulin with chitosan during the preparation process.

To prevent the microspheres from being degraded by stomach acid, we coated microspheres with cellulose acetate phthalate (CAP). 750 mg CAP was dissolved separately in a 30 mL mixture of ethylalcohol and DCM (ratio 2:1) to prepare a coating solution. The ZIF-8@OXA@inulin microspheres were dispersed in 100 mL distilled water and then mixed with coating solution and stirred at 1000 rpm. After complete evaporation of the solvent, 0.1M HCl solution was added dropwise to the ZIF-8@OXA@inulin and CAP mixture under stirring to adjust pH to 4.6. After stirring for 1 h, the dispersion medium was centrifuged to collect microspheres, and then washed microspheres three to four times with distilled water to remove the excess CAP. The coated microspheres were collected by centrifugation at 3,000 rpm for 10 min and then washed microspheres three times with distilled water to remove the excess CAP and stored in 1 mL 1% acetic acid solution. As a control, ZIF-8@OXA@Chitosan microspheres were coated by CAP during the preparation process. For brevity, ZIF-8@Chitosan@CAP and ZIF-8@OXA@Chitosan@CAP are hereafter referred to as ZIF-8@Chitosan and ZIF-8@OXA@Chitosan, respectively.

1.5 Material characterization.

The transmission electron microscopy (TEM) images samples were obtained using a FEI Tecnai G2 S-Twin with a field emission gun operating at 200 kV. Their morphology and size were observed by scanning electron microscope (SEM) of ZEISS Sigma 360. Their crystal structures were determined by X-ray powder diffraction (Bruker) equipped with Cu-Kα radiation (λ=0.154 nm). The X-ray photoelectron spectra (XPS) were taken on a VG ESCALAB MK II electron spectrometer using Mg Kα (1200 eV) as the excitation source, putting the samples on silicon slice. The concentrations of Zn ions were tested by inductively coupled plasma-mass spectrometer (ICP-MS) and ion chromatography. The mean particle sizes and zeta potential of ZIF-8@OXA@inulin microspheres were measured by a DLS analyzer (Malvern Zetasizer).

1.6 The degradation ability.

ZIF-8@OXA@inulin@CAP microspheres were dispersed in 5 mL phosphate-buffered saline adjusted to pH 3.0, 6.0, 7.4, or 8.0. Suspensions were incubated at 37 °C with gentle agitation (100 rpm) for 4 h. Particles were collected by centrifugation (3,000 rpm, 10 min), washed once with the corresponding buffer, air-dried, sputter-coated with gold, and imaged by SEM to evaluate morphology and size.

To further assess stability under physiologically relevant conditions, microspheres were incubated in simulated gastric fluid (SGF) and simulated colonic fluid (SCF). In a separate set, SCF was supplemented with *Bifidobacterium longum* at 1*10^6^ CFU/mL. At 0.5, 6, and 8 h, samples were harvested by centrifugation (3,000 rpm, 10 min), gently rinsed with the respective medium, air-dried, sputter-coated with gold, and examined by SEM. Morphological integrity, surface texture, and apparent particle size were compared across conditions and time points.

1.7 The release of OXA

Briefly, release was quantified indirectly by measuring Pt (as a proxy for the Pt(II) complex oxaliplatin) in the dialysate using ICP-MS. ZIF-8@OXA@inulin microspheres were placed in pre-wetted dialysis bags and incubated at 37 ℃ with gentle shaking (100 rpm) in 50 mL of simulated gastric fluid (SGF, pH 1.2) or simulated colonic fluid (SCF, pH 6.8). Besides, SCF was supplemented with *Bifidobacterium longum* at 1×10^6^ CFU/mL. At 0.5, 4, and 8 h, 4.0 mL of external medium was withdrawn, passed through a 0.22 µm filter, and analyzed by ICP-MS for Pt; an equal volume of fresh medium was then returned to maintain sink conditions and constant volume. The concentrations of Pt were converted to OXA equivalents using calibration curves prepared from OXA standards and the measured Pt content of the formulation. Cumulative release (%) was calculated.

1.8 Cell uptake and tumor homing effect of ZIF-8@OXA NPs

MC38 cells were collected and planted in confocal dish. 24 h later, MC38 cells were incubated with Rho-conjugated ZIF-8@OXA for 0, 2 h, 4 h and 8 h. Afterwards, the cells were fixed with 4% paraformaldehyde, sequentially stained with DAPI, and finally observed with a CLSM. Meanwhile, we further collected the treated cells and analyzed the intracellular fluorescence intensity of Rho through flow cytometry assay.

For TEM imaging, the treated cells were harvested and fixed with glutaraldehyde (2.5%), embedded in resin, sliced, stained by osmic acid, and finally the cell ultramorphology were observed with a TEM.

To assess the tumor homing effect of ZIF-8@OXA NPs in vivo, MC38 cells were collected and suspended with appropriate amount of PBS, and then 150 μL of suspension was injected onto the C57 mice to construct tumor-bearing mice models. Subsequently, the in vivo fluorescence images were captured. For in vivo fluorescence imaging, the tumor-bearing mice were intravenously injected Cy5.5 labeled ZIF-8@OXA NPs, which was imaged at different time periods (2h, 4h, 8h, 12h and 24h). Besides, in vivo fluorescence imaging of major organs were also captured at 12h.

1.9 Biological Evaluation *in vitro*

The Caco-2/HT29-MTX co-culture model of the gastrointestinal epithelium was cultured. Briefly, Caco-2 and HT29-MTX cells were counted by an automated cell counter (Cellometer Auto T4, Nexcelom Bioscience, Lawrence, MA, USA), mixed in a ratio of 9:1 (Caco-2: HT29-MTX) and seeded with a final density of 1 * 10^5^ cells/cm^2^ in each insert. Cells were cultured at 37 ℃, 5% CO_2_, and 95% humidity and allowed to grow for 17 days. The medium (300 µL in the apical chamber and 700 µL in the basolateral chamber) was refreshed every 2 days. Caco-2 and HT29-MTX co-cultures with TEER values > 300 Ω × cm^2^ which reached a plateau (~17 days after seeding) were used for further experiments. To detect the cell cytotoxicity of ^F127^ZIF-8 and ^F127^ZIF-8@OXA NPs for the Caco-2/HT29-MTX co-culture model, the ^F127^ZIF-8 and ^F127^ZIF-8@OXA NPs at different concentrations (0, 5, 10, 20, 40, 80, 120, 160, 240 μg/mL) was added and co-incubated for another 24 h. After that, cell viability was quantified with CCK-8 detection kit through a microplate reader. Additionally, the lactate dehydrogenase (LDH) production was also detected. Besides, to detect the cell cytotoxicity of ^F127^ZIF-8 and ^F127^ZIF-8@OXA NPs for immune cells, we firstly seeded the cells (RAW264.7, mouse bone marrow neutrophils and CD8+ T cells) in 96-well plates. 24 h later, the ^F127^ZIF-8 and ^F127^ZIF-8@OXA NPs at different concentrations ([ZIF-8]=0, 5, 10, 20, 40, 80, 120, 160, 240 μg/mL) were added and co-incubated for another 24 h. After that, cell viability was quantified with CCK-8 detection kit.

Tight-junction formation in the Caco-2/HT29-MTX co-culture was verified by western blotting of ZO-1 and occludin. Briefly, the total proteins of treated cells were extracted with a lysis buffer containing protease inhibitors and phosphatase inhibitors. Subsequently, the target proteins were separated by SDS-polyacrylamide gel and transferred onto PVDF membrane. Afterwards, these membranes were blocked with 5% BSA, followed by incubated with anti-ZO-1 and anti-occludin and secondary antibodies. Finally, the protein bands were visualized with an enhanced chemiluminescence detection system.

Hemolysis assay of red blood cells (RBCs) was carried out to explore the blood compatibility of ^F127^ZIF-8@OXA NPs. Firstly, fresh blood sample was collected from eye venous sinus of SD rats, and the RBCs were isolated from serum by centrifugation, and washed three times with sterile normal saline. Then, RBCs were diluted to a concentration of 2% (v/v) solution by sterile normal PBS and were separately mixed with an equal volume of ^F127^ZIF-8@OXA NPs solutions at different concentrations (20, 40, 80, 160, 320, 500 and 1000 μg/mL). Herein, RBC diluted with TritonX-100 and PBS were acted as the positive and negative controls, respectively. After standing at room temperature for 4 h, all the sample tubes were centrifuged at 8000 rpm for 5 min, the hemolytic photographs of RBCs were taken and the absorbances of the supernatants were detected using ultraviolet spectroscopy.

1.10 Establishment of Mouse Orthotopic Colon Tumor Models

Female C57 mice (6−8 weeks) were purchased from Medical Experimental Animal Center of Guangdong Province (Guangdong, China). All animal procedures were approved by Nanfang Hospital Experimental Animal Ethics Committee (IACUC-LAC-20231219-002), and all investigation procedures were carried out in accordance with the Helsinki Declaration. Mice were intraperitoneally injected with the AOM solution (10 mg/kg). Seven days after injection, mice were subjected to two cycles of DSS treatment, with each (2.5%, w/v) for 7 days, followed by a 14-day recovery period with regular water, which were ready for the following experiments.

1.11 Biodistribution

Firstly, in order to evaluate the in vivo bio-distribution of ZIF-8@OXA NPs, the healthy mice were intravenously injected with ZIF-8@OXA NPs (dose = 20 mg/kg, 150 μL). At the indicated time point (2 h, 8 h, 12 h, 24 h), the main organs and tumor were collected, weighted and dissolved in digesting aqua regia. The concentrations of Zn ions in different samples were detected by ICP-MS.

To explore the distribution of ZIF-8@OXA@inulin microspheres in vivo, ZIF-8@OXA@inulin microspheres-ICG was prepared by the same way as ZIF-8@OXA@inulin microspheres. After the mouse orthotopic colon tumor models was established, the mice were randomly divided into two groups (n = 3 per group) and orally administered with free ICG or ZIF-8@OXA@inulin microspheres-ICG (ICG concentration: 2 mg/kg). After gaseous anesthesia, all mice were photographed by IVIS (IVIS Spectrum, Perkin Elmer, USA) at certain time points (2, 4, 8, and 12 h). Images were analyzed using IVIS Living Image Software.

1.12 In vitro cytotoxicity studies

In order to detect the cell cytotoxicity of ZIF-8@OXA NPs for MC38 cells, we firstly seeded the cells in 96-well plates. 24 h later, the DMEM containing ZIF-8 and ZIF-8@OXA NPs at different concentrations ([ZIF-8]=0, 5, 10, 20, 40, 80, 120, 160, 240 μg/mL) was added and co-incubated for another 24 h with and without MCC950 (10 μM). After that, cell viability was quantified with CCK-8 detection kit through a microplate reader.

For the Live/Dead staining assay, the MC38 cells were seeded in 24-well plates and cultured overnight. Subsequently, different NPs ([ZIF-8]=80 μg/mL) were added and co-incubated for another 24 h with and without MCC950 (10 μM). Afterwards, the treated cells were washed with PBS solution, and stained with Calcein-AM and PI probe, and finally observed via inverted fluorescence microscopy. Besides, the treated cells were further collected and incubated with 7-AAD probes, and finally the results were analyzed by flow cytometry.

1.13 Intracellular Zn ions detection.

Intracellular Zn ions detection was examined by a Zn ions probe [N-(6-Methoxy-8-quinolyl)-p-toluenesulfonamide] (TSQ, AAT Bioquest, Inc.). MC38 cells were seeded into 12-well plates at the density of 1×10^5^ cells per well and then incubated with Control, ZIF-8 and ZIF-8@OXA NPs ([ZIF-8] = 80 μg/mL) for 4 h, respectively. Furthermore, different concentrations of ZIF-8 and ZIF-8@OXA NPs (0, 20, 40, 80 μg/mL) was also incubated. Meanwhile, ROS scavenger N-acetylcysteine was also administered. Then 1 μL of TSQ DMSO solution (0.1 M) was added and then was incubated for 30 min at 37 ℃. Next, the culture media were replaced and washed by fresh PBS and then observed under inverted fluorescence microscope. Meanwhile, the cells were also analyzed by flow cytometry.

1.14 Detection of mitochondrial function

Firstly, the MC38 cells were collected and planted in 24-well plate for 24 hours. Subsequently, the cells were treated with ZIF-8 and ZIF-8@OXA NPs ([ZIF-8]=80 μg/mL) for another 24 hours. Afterwards, the treated cells were collected and stained with DCFH-DA probe, JC-1 mitochondrial membrane potential assay kit, mPTP detection kit, and MitoSOX^TM^ red mitochondrial superoxide indicator, to determine the intracellular ROS level, mitochondrial membrane potential level, mPTP opening level, and mitoROS level, respectively. Additionally, the treated cells were harvested and fixed with glutaraldehyde (2.5%), embedded in resin, sliced, stained by osmic acid, and finally the cell ultra-morphology were observed with a TEM.

1.15 Dection of Nuclear/Mitochondrial DNA Dual Damage

First, MC38 cells were seeded in CLSM dishes and cultured overnight. Second, ZIF-8 and ZIF-8@OXA NPs ([ZIF-8]=80 μg/mL) were added and incubation. After 24 h, the cells were fixed with 4% paraformaldehyde for 10 min and permeabilized in 0.3% Triton X-100 solution for 10 min. Then, the treated cells were incubated with both 8-OHdG antibody and TOMM20 antibody for the detection of oxidized mitoDNA. After 12 h, the treated cells were incubated with secondary antibody and finally stained with DAPI. Images were captured by CLSM. Additionally, the treated cells were incubated with γ-H2AX antibody for the detection of damaged nuclear DNA.

1.16 Detection of intracellular HIF-1α expression after various treatment

Firstly, the MC38 cells were collected and planted in confocal dishes for 24 h. Subsequently, the cells were co-incubated with various nanocomposites ([ZIF-8]=80 μg/mL). 24 h later, the cells were washed with PBS, fixed with 4% paraformaldehyde. After blocked with 5% BSA, the cells were sequentially stained with HIF-1α antibody and secondary fluorescent antibodies. Finally, the represented images were captured by CLSM.

1.17 Western blot analysis of Pyroptosis

After extracting tumor tissues or cells with protein lysate, equal amounts of protein were separated by sodium dodecyl sulfate poly-acrylamide gel electrophoresis (8–12% acrylamide gel) and subsequently transferred to polyvinylidene difluoride (PVDF) membranes. The PVDF membranes were blocked with 5% BSA and incubated with NLRP3, Caspase-1, Cleaved Caspase-1, GSDMD-NT, and GAPDH antibodies overnight at 4 ℃. Subsequently, the membranes were washed with PBST and incubated with goat anti-rabbit or anti-mouse IgG H&L (HRP; 1:2000, Abcam Co., USA) for 1 h at room temperature. Finally, the protein bands were visualized with an enhanced chemiluminescence detection system (Tianneng, China).

Furthermore, the supernatant from each cell sample after various treatment, were obtained to detect the release of IL-1β with ELISA kit. Additionally, the release of LDH (lactate dehydrogenase) was determined by the LDH detection kit (Roche Diagnostics).

1.18 Detection of immunogenic cell death biomarkers

MC38 cells were seeded in the confocal dishes for 24 h. Afterwards, the cells were treated with treated with ZIF-8 and ZIF-8@OXA NPs ([ZIF-8]=80 μg/mL). 24 h later, the treated cells were fixed with 4% paraformaldehyde for 15 min and blocked with 5% BSA for 1 h at room temperature. Subsequently, these cells were incubated with primary rabbit anti-CRT or anti-HMGB1 for 12 h at 4℃, followed by incubated with an FITC or Cy3-conjugated goat anti-rabbit IgG antibody for another 1 h at 4℃. Finally, the cells were further stained with DAPI before visualized under CLSM.

Additionally, the treated cells were collected to analyze the expression of CRT and HMGB1 protein through western blot and flow cytometry analysis. Furthermore, the supernatant from each cell sample after various treatment, were obtained to detect the release of HMGB1 with ELISA kit. Additionally, the release of ATP was determined with the chemiluminescence ATP determination kit.

1.19 *In vitro* macrophage polarization detection

Firstly, RAW 264.7 macrophages were cultured with IL-4 (25 ng/mL) for 12 h to induce M2 polarization. Subsequently, the MC38 cells were pre-treated with PBS, ZIF-8 and ZIF-8@OXA NPs ([ZIF-8] = 80 μg/mL); 24 h later, the corresponding cell supernatants were collected through centrifugation. Afterwards, the MC38 cell supernatants and PBS were added and co-incubated with M2 macrophages for another 12 h. Afterwards, RAW 264.7 macrophages were collected and stained by anti-CD86 and anti-CD206 and then was measured by CLSM or flow cytometry.

1.20 *In vitro* DCs maturation detection

MC38 cells were exposed to ZIF-8 or ZIF-8@OXA ([ZIF-8]=80 μg/mL). To evaluate the contribution of pyroptosis to DC maturation, the NLRP3 inhibitor MCC950 (10 µM) was included where indicated. Specifically, MC38 cells were treated with ZIF-8@OXA in the presence or absence of MCC950, after which the treated MC38 cells were co-cultured with bone-marrow–derived dendritic cells (BMDCs) for 24 h in a Transwell system. BMDCs were then collected and stained with anti-CD80 and anti-CD86 for flow-cytometric analysis of maturation. For immunostaining, BMDCs from the control, ZIF-8, and ZIF-8@OXA groups were blocked with BSA, sequentially incubated with primary antibodies (anti-CD80 and anti-CD86) and appropriate fluorophore-conjugated secondary antibodies, and examined by confocal laser scanning microscopy (CLSM) or analyzed by flow cytometry.

1.21 In vitro NETs formation detection

Mouse bone marrow neutrophils (from C57BL/6 mice) were isolated using MACS mouse neutrophil isolation kit (Miltenyi Biotec). The MC38 cells were treated with ZIF-8 and ZIF-8@OXA NPs ([ZIF-8] = 80 μg/mL), respectively. To evaluate the contribution of pyroptosis to NETs formation, the NLRP3 inhibitor MCC950 (10 µM) was included where indicated.

Afterwards, the treated cells were then co-incubated with the neutrophils for 24 h using a Transwell system. Cells were blocked with BSA and stained with DAPI, histone 3 and myeloperoxidase antibody to detect NETs. Sequentially, cells were stained with secondary fluorescent antibodies and finally observed through CLSM.

1.22 Detection of intracellular lipid peroxidation (LPO) level in neutrophils

Briefly, the MC38 cells were treated with ZIF-8 and ZIF-8@OXA NPs ([ZIF-8] = 80 μg/mL), respectively. Afterwards, the treated cells were then co-incubated with the neutrophils for 24 h using a Transwell system. Afterward, the cells were stained with BODIPY 581/591 C11 probe for 30 min, followed by with Hochest 33342. Finally, the signals from reduced C11 (PE channel) and oxidized C11 (FITC channel) were monitored via CLSM and flow cytometry assay. Additionally, the release of TGF-β was measured by an ELISA kit from BioSource International.

1.23 Anti-tumor effect

Orthotopic colon tumor models mice were randomly divided into 7 groups (n = 6 per group): (i) saline, (ii) chitosan, (iii) ZIF-8@Chitosan, (iv) ZIF-8@OXA@Chitosan, (v) inulin, (vi) ZIF-8@inulin, (vii) ZIF-8@OXA@inulin. Mice were orally treated with various formulations (20mg/kg OXA; 250mg/kg Chitosan or inulin) every 3 days. The body weights were measured every 4 d. On the 18 day, mice from each group were suffocated to death, and their tumors and intact intestines were taken out. The intestines were sectioned and stained with H&E, N-cadherin and Ki67.

Furthermore, orthotopic colon tumor models mice were randomly divided into 4 groups (n = 6 per group): (i) saline, (ii) MCC950, (iii) ZIF-8@OXA@Chitosan, (iv) ZIF-8@OXA@Chitosan + MCC950 (20mg/kg OXA; 250mg/kg Chitosan or inulin) every 3 days. On the 18 day, mice from each group were suffocated to death, and their tumors and intact intestines were taken out. The intestines were sectioned and stained with H&E. NLRP3 inhibitor MCC950 (10 mg/kg) was intraperitoneally injected 30 min before ZIF-8@OXA@Chitosan treatment.

To examine the immune response caused by ZIF-8@OXA@inulin, the spleen, tumor-draining lymph node, and tumor tissues were surgically resected from mice in different groups. After digesting with collagenase IV (0.3 mg/mL) at 37°C for 1 h, the single cell suspensions could be collected via filtration with a 70 μm mesh. Subsequently, the collected cells were blocked with CD16/CD32 antibody for 15 min, sequentially with eBioscience^TM^ Fixable Viability Dye eFluor^TM^ 506 for 15 min at 4℃.

The collected cells were then incubated with anti-CD45, anti-CD3, and anti-CD8 antibodies to evaluate the content of CD8^+^ T cells in the tumors using flow cytometry, following standard protocols. Additionally, the effective CD8^+^ T cells in the tumors were identified with anti-CD45, anti-CD3, and anti-CD8 antibodies. Then, the collected cells were stained with anti-CD45, anti-CD3, anti-CD4, and anti-Foxp3 antibodies to evaluate the proportion of Treg cells. Subsequently, the collected cells were stained with anti-CD45, anti-CD11b, anti-F4/80, anti-CD206 and anti-CD86 antibodies for M1/M2 detection. To detect the neutrophils in the tumor, the collected cells were stained with anti-CD45, anti-CD11b, anti-Ly6G, and antibodies. Additionally, the frequency of mature DCs in the lymph nodes was examined by flow cytometry after staining with anti-CD45, anti-CD11c, anti-CD80 and anti-CD86.

1.24 Assessment of lung metastasis inhibition

To evaluate the effect of ZIF-8@OXA@inulin on lung metastasis inhibition, C57 mice (female, 4 weeks old) were injected with MC38 cells via tail vein injection. After a 7-day incubation, the lung metastatic models were built by injecting MC38 cells (10^6^ cells, 100 μL) through the caudal vein. Then the mice were randomly divided into groups as follows (n=6): (i) saline, (ii) Chitosan, (iii) ZIF-8@Chitosan, (iv) ZIF-8@OXA@Chitosan, (v) inulin, (vi) ZIF-8@inulin, (vii) ZIF-8@OXA@inulin. On day 18, the mice were sacrificed, and the lungs were dissected and fixed for HE stains.

1.25 Gut Microbiota Analyses

Orthotopic colon tumor models mice were randomly divided into 7 groups (n = 6): (i) saline, (ii) Chitosan, (iii) ZIF-8@Chitosan, (iv) ZIF-8@OXA@Chitosan, (v) inulin, (vi) ZIF-8@inulin, (vii) ZIF-8@OXA@inulin. Mice were orally treated with various formulations (20mg/kg OXA; 250mg/kg Chitosan or inulin) every 3 days. On the 18st, feces of each group were collected quick-frozen in liquid nitrogen for 30 min, and stored at -80℃. Microbiome DNA isolation and 16 S rDNA gene sequencing were completed with the help of Majorbio Co. Ltd., Shanghai. Isolation of microbial DNA from the feces of the mice was performed using a Qiagen E. Z.N. A.® Soil DNA Kit. The V3-V4 region of the 16 S rRNAencoding gene was amplified from extracted DNA using the barcoded dual-index primers. The PCR product was identified by gel electrophoresis, purified by AxyPrep DNA Gel Extraction Kit and quantified by Quantus^TM^ Fluorometer. The pooled amplicon library was then sequenced on the Illumina MiSeq platform using the NEXTFLEX Rapid DNA-Seq Kit according to the manufacturer’s instructions. The raw data was processed on the Qiime2 (version 2022.2), including reducing sequencing and PCR errors, and denoising by DADA2 (filtering, dereplication, chimera identification, and merging paired end reads and so on) to optimize the sequence and get amplicon sequence variants (ASVs) for taxonomic analysis. ASVs fewer than 0.1% in all the samples or annotated as chloroplast and mitochondrial contaminants were removed from all the samples. The sequencing numbers in all samples were normalized to minimal values. Based on the Silva 138/16 s bacteria database, species taxonomic analysis of ASVs was performed using the Naive bayes classifier. Alpha-diversity and beta diversity index was calculated by monther software (version 1.30). The intestine microbial abundance in healthy C57 mice was also analyzed.

1.26 Potent antitumour of ZIF-8@OXA NPs-embedded inulin microspheres plus α-PD-1 therapy with and without clearance of gut microbiota.

Orthotopic colon tumor models mice were randomly divided into 9 groups (n = 6): (i) saline, (ii) αPD-1, (iii) ZIF-8@OXA@inulin, (iv) inulin+αPD-1, (v) ZIF-8@OXA@inulin+αPD-1, (vi) αPD-1+ABX, (vii) ZIF-8@OXA@inulin+ABX, (viii) inulin+ABX+αPD-1, (ix) ZIF-8@OXA@inulin+ABX+αPD-1. Mice were orally treated with various formulations (20mg/kg OXA; 250mg/kg Chitosan or inulin) every 3 day. The body weights were measured every 4 days. On the 18 day, mice from each group were suffocated to death, and their tumors and intact intestines were taken out. The intestines were sectioned and stained with H&E and Ki67.

The collected cells were then incubated with anti-CD45, anti-CD3, and anti-CD8 antibodies to evaluate the content of CD8^+^ T cells in the tumors using flow cytometry, following standard protocols. Additionally, the effective CD8^+^ T cells in the tumors was identified with anti-CD45, anti-CD3 and anti-CD8. Then, the collected cells were stained with anti-CD45, anti-CD3, anti-CD4, and anti-Foxp3 antibodies to evaluate the proportion of Treg cells. To detect the neutrophils in the tumor, the collected cells were stained with anti-CD45, anti-CD11b, anti-Ly6G antibodies.

For paraffin-embedded tissue, the instine were fixed overnight at 4℃ in 4% PFA, rinsed with PBS and transferred into 70% ethanol, processed using conventional methods, embedded in paraffin, and sectioned at 8 mm. Paraffin-embedded tissue sections were deparaffinized and rehydrated, and antigen retrieval was performed in EDTA buffer (10 mM Tris Base, 1 mM EDTA solution, 0.05% Tween 20, pH=9.0). Sections were blocked with Fc Receptor blocker and incubated with 1X blocking buffer (5% donkey serum, 2.5% BSA, 0.1% Triton X-100 in PBS). Then, sections were incubated overnight at 4℃ with anti-myeloperoxidase (1:100) and anti-citrullinated histone H3 antibodies (1:250) in 0.5X blocking buffer. After three washes with PBS, the sections were incubated with the suitable fluorochrome-conjugated secondary antibodies (1:150) in 0.5X blocking buffer for 45 min in the dark at room temperature. After two washes with PBS and one with water, sections were counterstained with DAPI and rinsed in water, and the slides were mounted onto coverslips using mounting media.

1.27 Statistical analysis

Data is shown as mean ± standard deviation (SD). Quantitative analysis of the immunofluorescence intensity was conducted using the Image J program (National Institutes of Health, Bethesda, MD). Statistical analysis statistics were performed using GraphPad Prism 8. Differences between two experimental groups were determined by two-tailed Student’s t-test and multiple groups by one-way ANOVA with Tukey’s post-test. * indicates *p* < 0.05; ** indicates *p* < 0.01; *** indicates *p* < 0.001.

1. Supplementary Figures


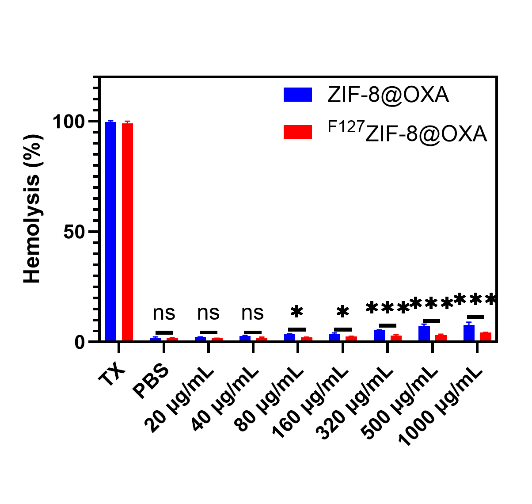


**Figure S1**. Hemolysis test of RBCs in TritonX-100, PBS, and NPs at various concentrations (n=3). * *p* <0.05, ** *p* <0.01, *** *p* <0.001


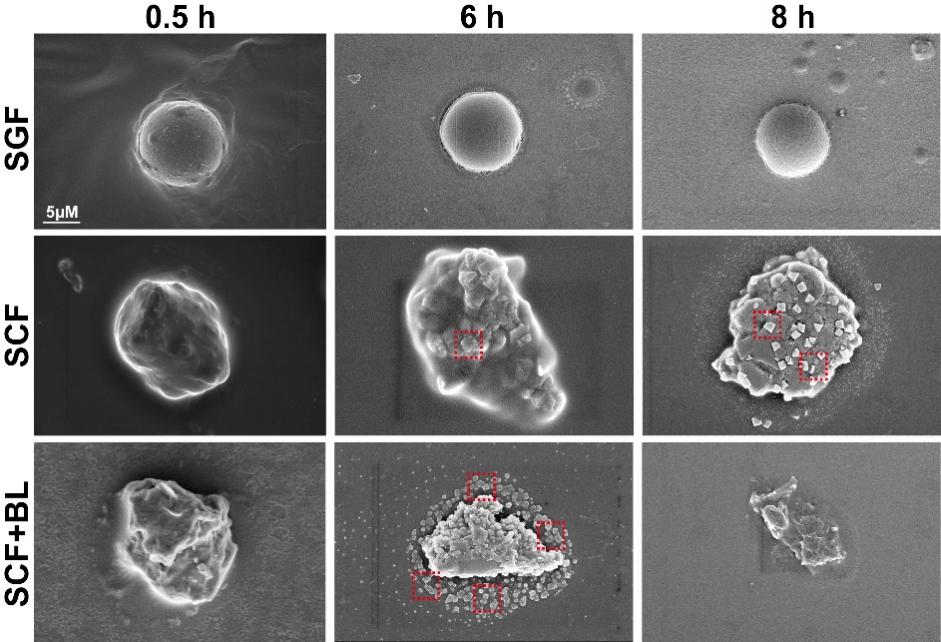


**Figure S2**. SEM image of ZIF-8@OXA@inulin@CAP microspheres when being exposed to SGF, SCF and SCF with *Bifidobacterium longum* under different time point. Scale bar: 5 μM.


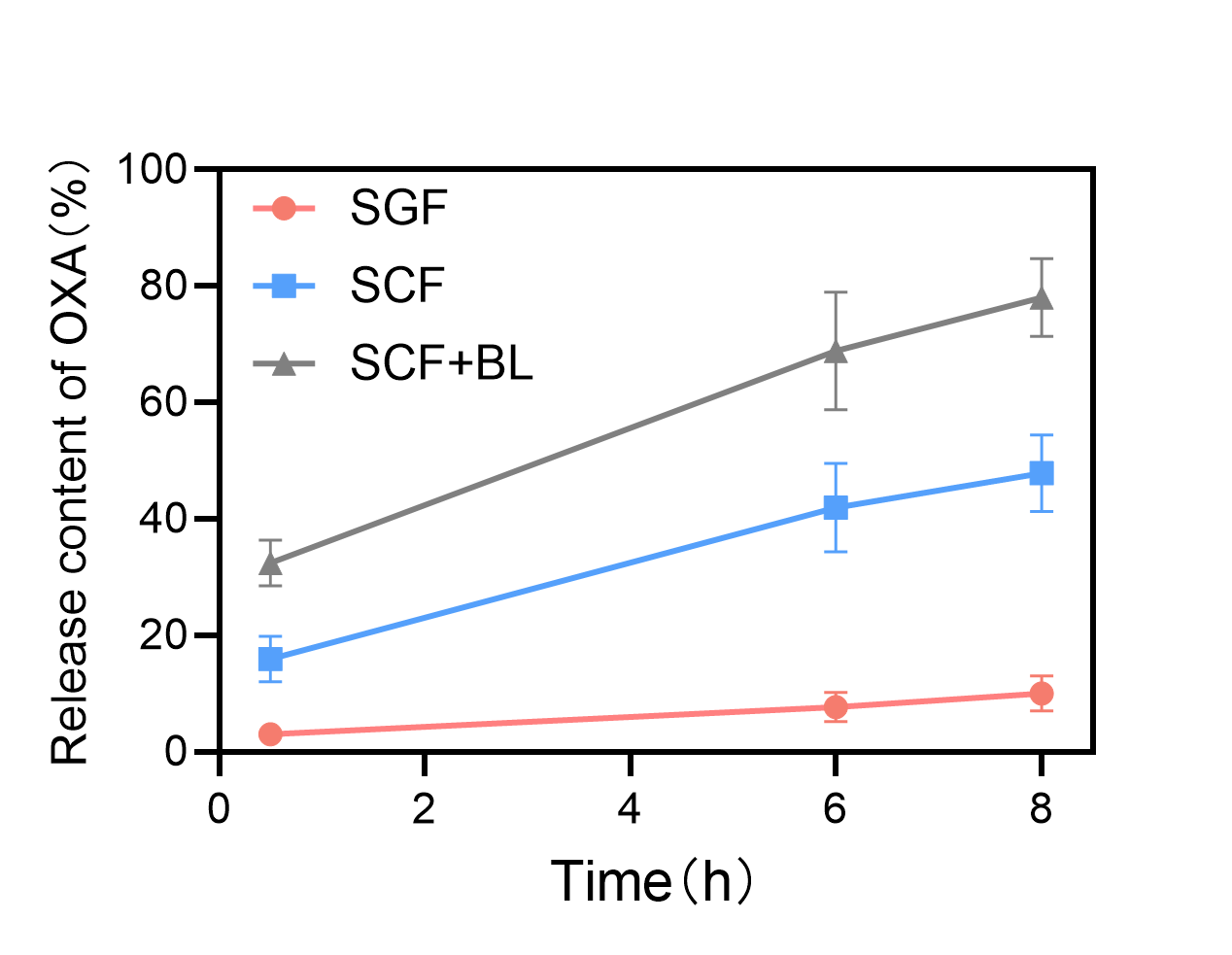


**Figure S3**. OXA release profiles of ZIF-8@OXA@inulin@CAP microspheres when being exposed to SGF, SCF and SCF with *Bifidobacterium longum* under different time point.


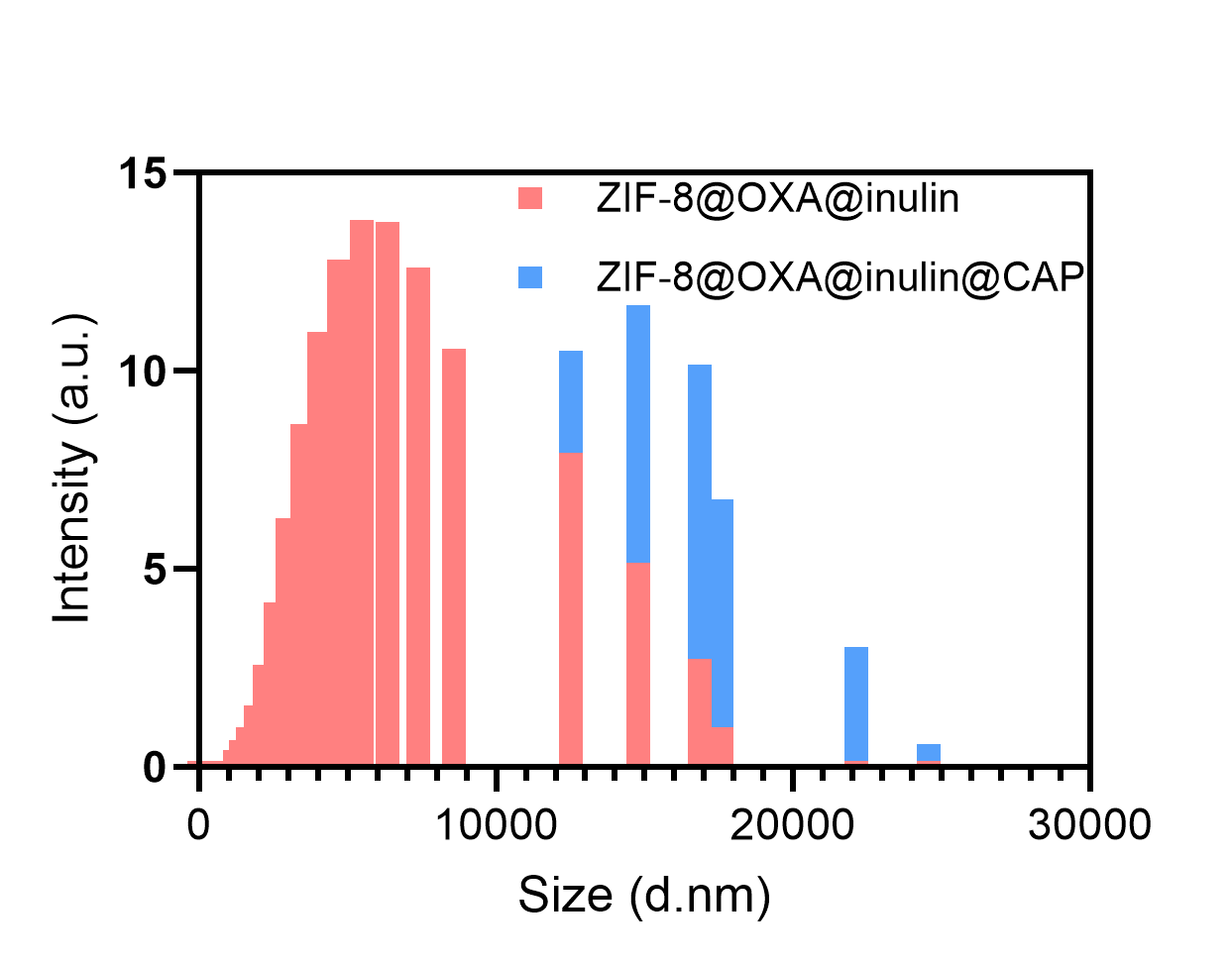


**Figure S4.** Hydrodynamic size distribution of microspheres.


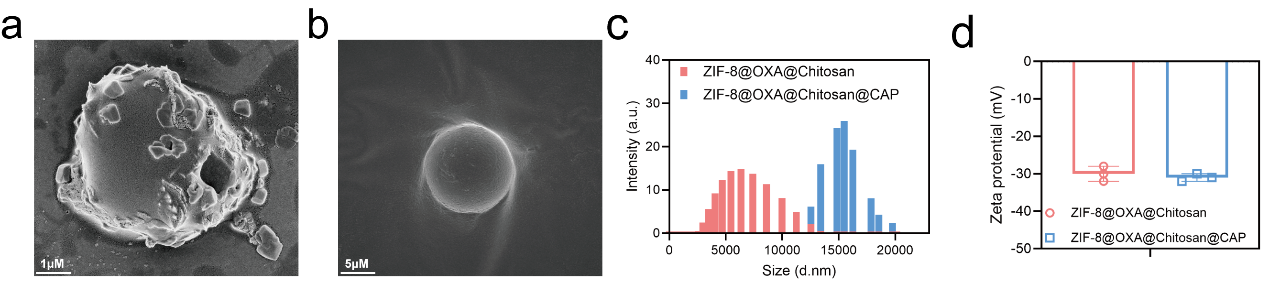


**Figure S5**. (a-b) SEM image of ZIF-8@OXA@Chitosan (a) and ZIF-8@OXA@Chitosan@CAP microspheres (b). (c) Hydrodynamic size distribution of microspheres. (d) Zeta potential (mV) of microspheres (n = 3).


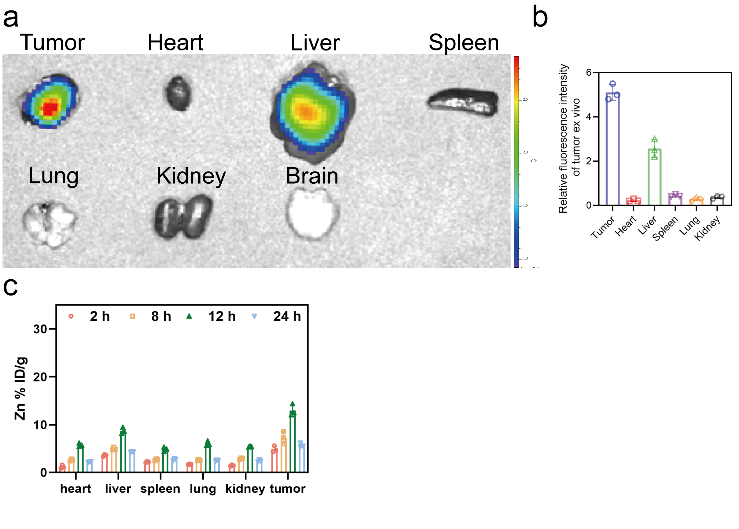


**Figure S6**. (a) Fluorescence images of major organs from the mice after 12 h post-injection. (b) Relative fluorescence intensity for the ex vivo images. (c) Zn concentration in the major organs (heart, liver, spleen, lung, kidney) and tumor detected by ICP-MS after i.v. injected with ZIF-8@OXA NPs (dose = 20 mg/kg, 150 μL) for different time (n=3). Data are presented as mean ± SD.


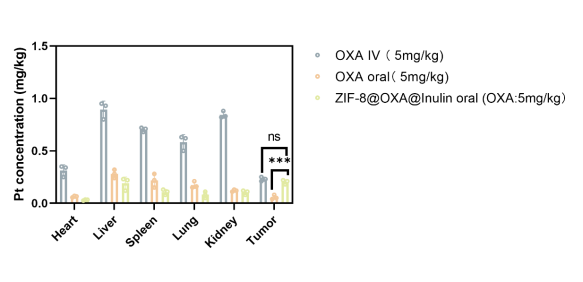


**Figure S7.** The content of OXA in different organs after the IV or oral administration of free OXA or ZIF-8@OXA@Inulin microspheres in orthotopic colon tumor model mice (n = 3). * *p* < 0.05, ** *p* < 0.01, *** *p* < 0.001.


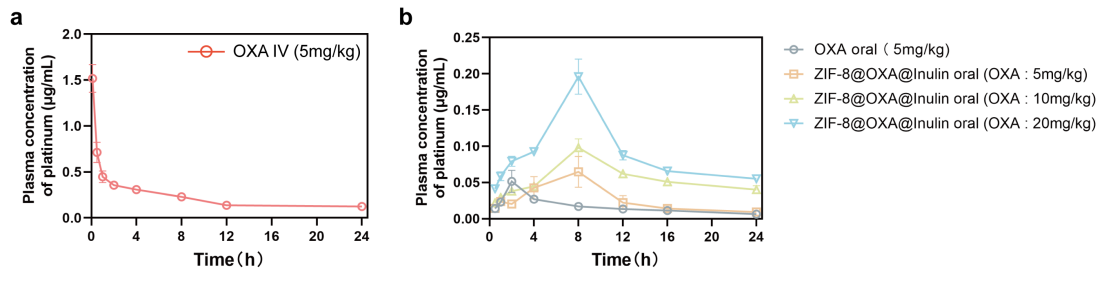


**Figure S8.** Plasma concentration-time curves of OXA after the IV or oral administration of free OXA or ZIF-8@OXA@Inulin microspheres in mice (n = 3).


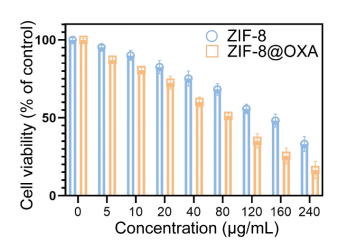


**Figure S9.** Viability of MC38 cells after being treated with ZIF-8 or ZIF-8@OXA NPs at different concentrations (n = 3).


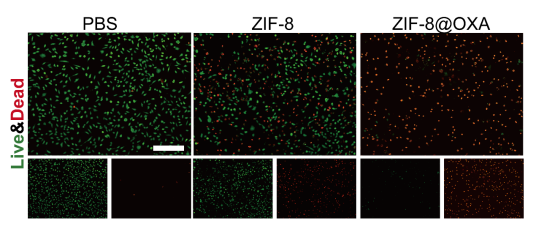


**Figure S10.** Live/Dead cell assay of treated MC38 cells. Scale bars: 100 μm.


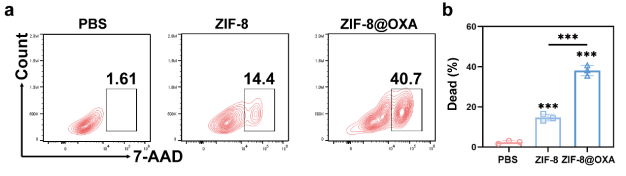


**Figure S11.** The proportion of 7-AAD+ cells analyzed by flow cytometry (n = 3). * *p* < 0.05, ** *p* < 0.01, *** *p* < 0.001.


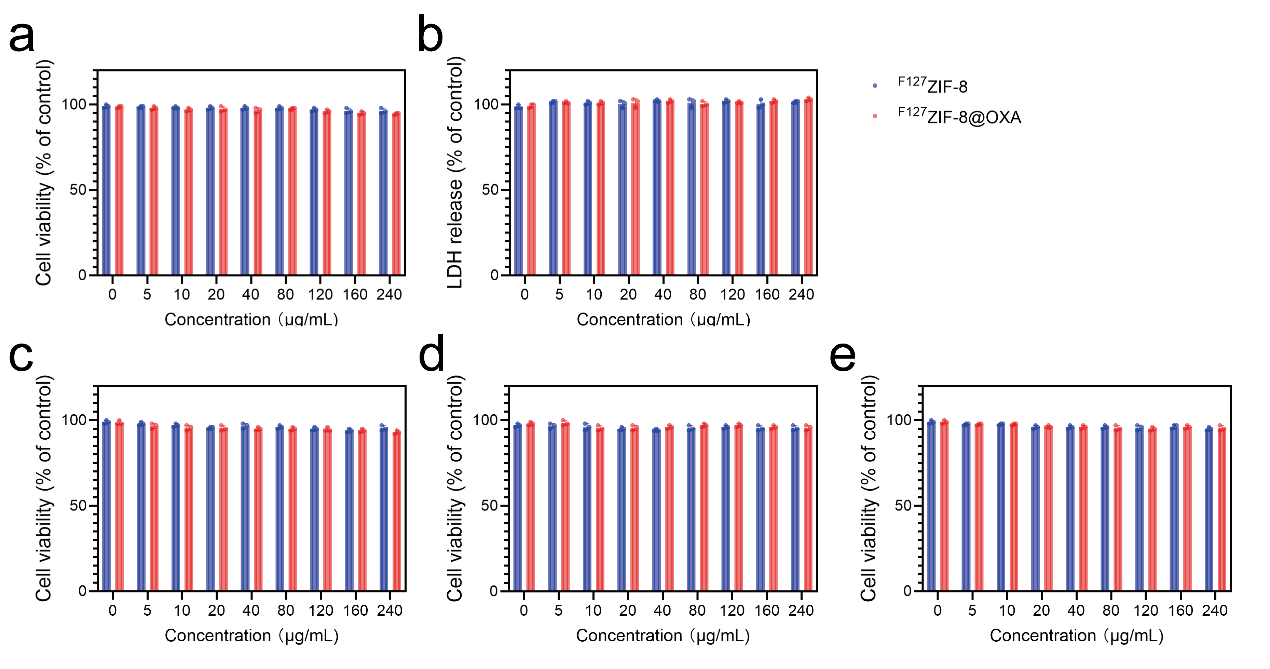


**Figure S12**. (a) Viability after being treated with ^F127^ZIF-8 or ^F127^ZIF-8@OXA NPs at different concentrations (n = 3) in Caco-2/HT29-MTX co-culture model. (b) Lactate dehydrogenase (LDH) production detection after being treated with ^F127^ZIF-8 or ^F127^ZIF-8@OXA NPs at different concentrations (n = 3) in Caco-2/HT29-MTX co-culture model. (c-e) Viability after being treated with ^F127^ZIF-8 or ^F127^ZIF-8@OXA NPs in RAW264.7 (c), mouse bone marrow neutrophils (d) and CD8+ T cells (e).


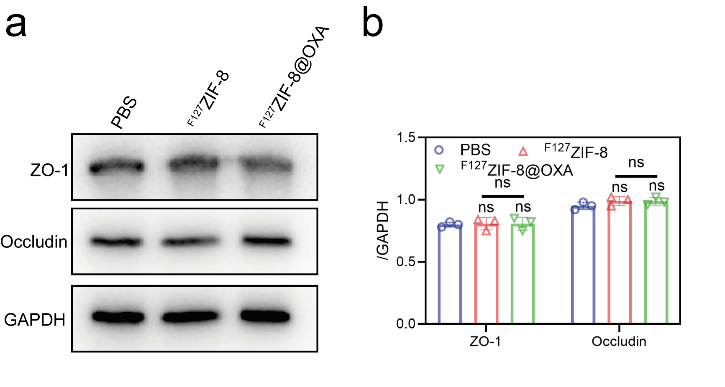


**Figure S13**. (a) Western blotting results and (b) statistical analysis of ZO-1, Occludin, and GAPDH expression in Caco-2/HT29-MTX co-culture model after treatment (n = 3). * *p* < 0.05, ** *p* < 0.01, *** *p* < 0.001.


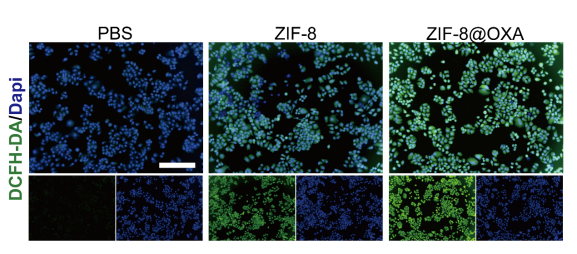


**Figure S14.** The fluorescence images of intracellular ROS level stained with DCFH-DA probe (Scale bar, 200 μm).


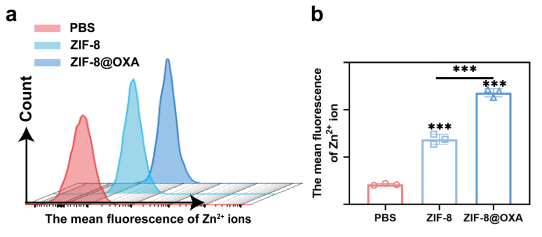


**Figure S15.** (a) FCM of the Zn^2+^ ion production in MC38 cell after incubation with ZIF-8 or ZIF-8@OXA NPs. (b) Corresponding quantification of mean fluorescence intensity inside cells (n = 3). * *p* < 0.05, ** *p* < 0.01, *** *p* < 0.001.


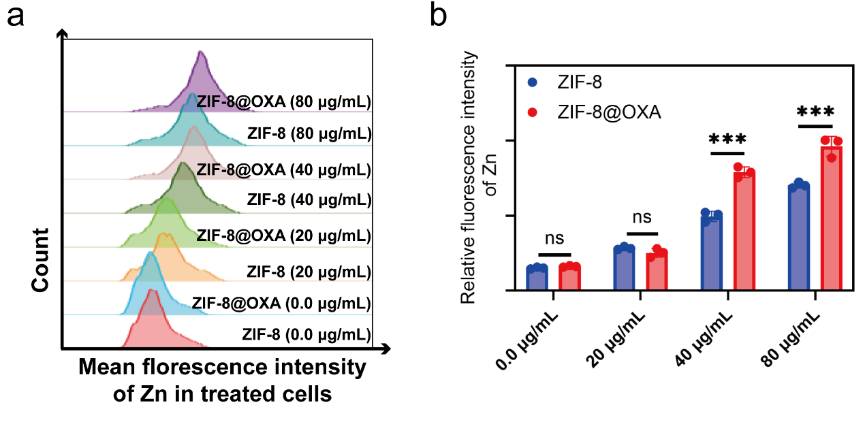


**Figure S16**. (a) FCM of the Zn^2+^ ion production in MC38 cell after incubation with ZIF-8 or ZIF-8@OXA NPs at different concentration. (b) Corresponding quantification of mean fluorescence intensity inside cells (n = 3). * *p* <0.05, ** *p* <0.01, *** *p* <0.001.

**
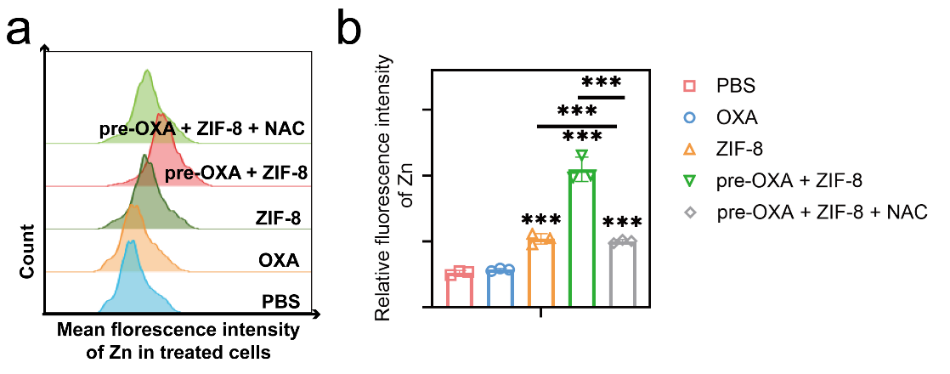
**

**Figure S17**. (a) FCM of the Zn^2+^ ion production in MC38 cell after different treatment. (b) Corresponding quantification of mean fluorescence intensity in cells (n = 3). * *p* <0.05, ** *p* <0.01, *** *p* <0.001.


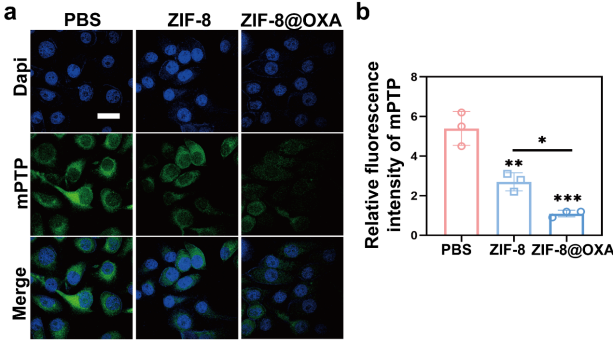


**Figure S18**. (a) Intracellular mPTP level and (b) statistical analysis of treated MC38 cells (n = 3), scale bars:20 μm. * *p* < 0.05, ** *p* < 0.01, *** *p* < 0.001.


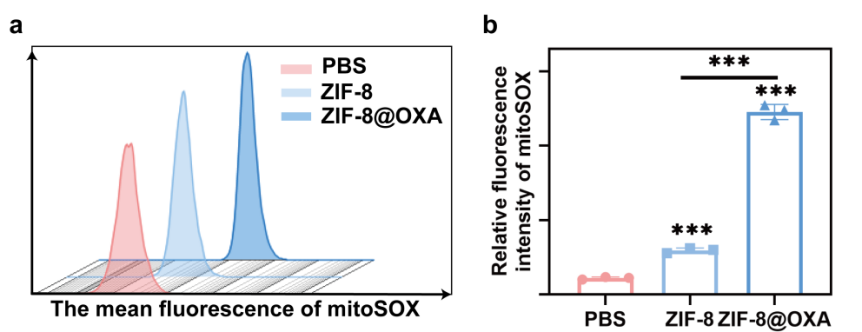


**Figure S19**. (a) Flow cytometric analysis of intracellular mitoROS level. (b) Statistical analysis of relative mean mitoROS level inside cells (n = 3). * *p* < 0.05, ** *p* < 0.01, *** *p* < 0.001.


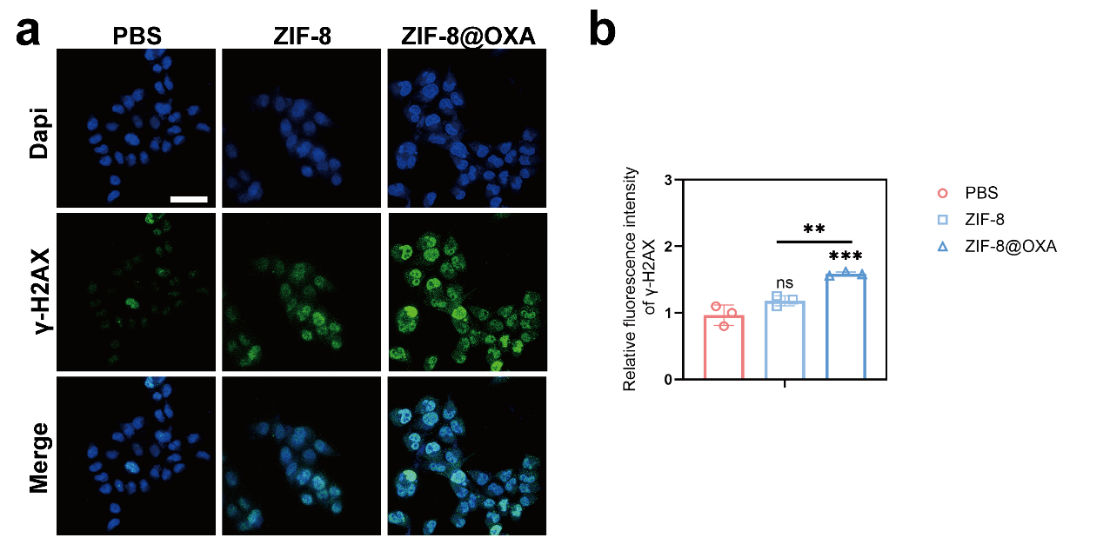


**Figure S20**. (a) γ-H2AX immunostaining applied to evaluate nDNA oxidation levels in treated MC38 cell, Scale bar: 30 μm. (b) Statistical analysis of relative fluorescence intensity of γ-H2AX (n = 3). **p* < 0.05, ** *p* < 0.01, *** *p* < 0.001.


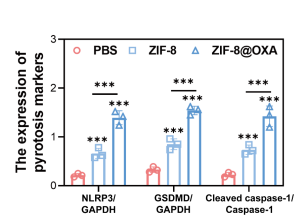


**Figure S21**. Western blot statistical analysis of NLRP3, GSDMD and Cleaved caspase-1 proteins expression in MC38 cells after treatment (n = 3). * *p* < 0.05, ** *p* < 0.01, *** *p* < 0.001.


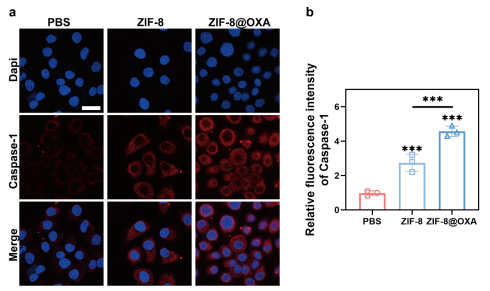


**Figure S22**. (a) Intracellular caspase-1 level and (b) statistical analysis of treated MC38 cells, scale bars:20 μm (n = 3). * *p* < 0.05, ** *p* < 0.01, *** *p* < 0.001.


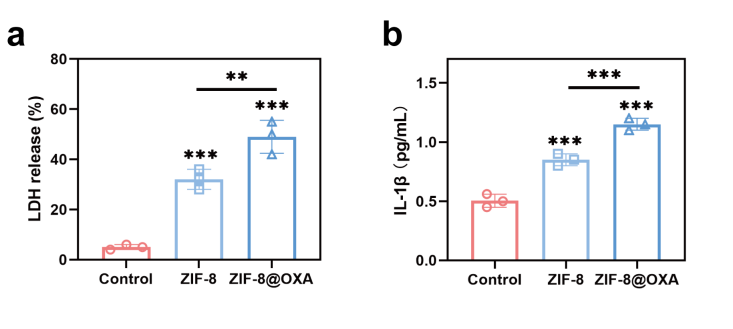


**Figure S23**. (a) The relative amounts of released lactic dehydrogenase (LDH). (b) The amounts of released intereukin-1β (IL-1β) (n = 3). * *p* < 0.05, ** *p* < 0.01, *** *p* < 0.001.


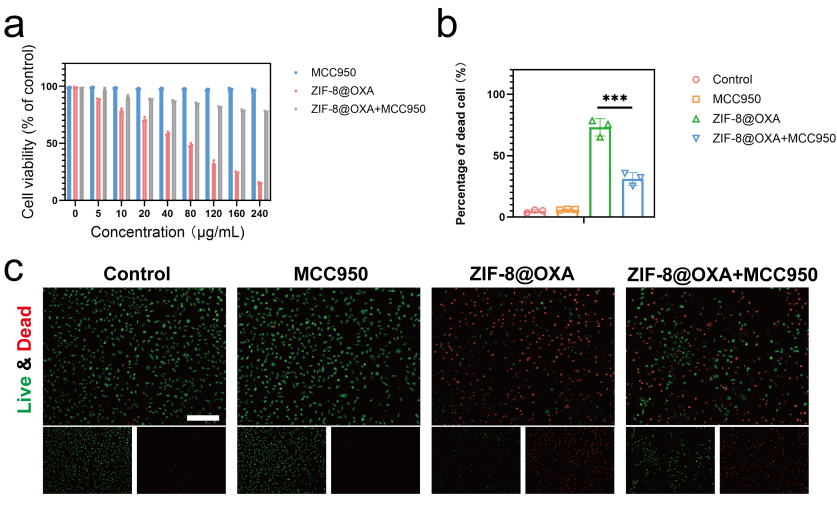


**Figure S24**. (a) Viability of MC38 cells after being treated with MCC950 at different concentrations. Data represent the mean ± SD (n = 3). (b-c) Live/Dead cell assay and statistical analysis of treated MC38 cells. Scale bars: 100 μm. * *p* < 0.05, ** *p* < 0.01, *** *p* < 0.001.


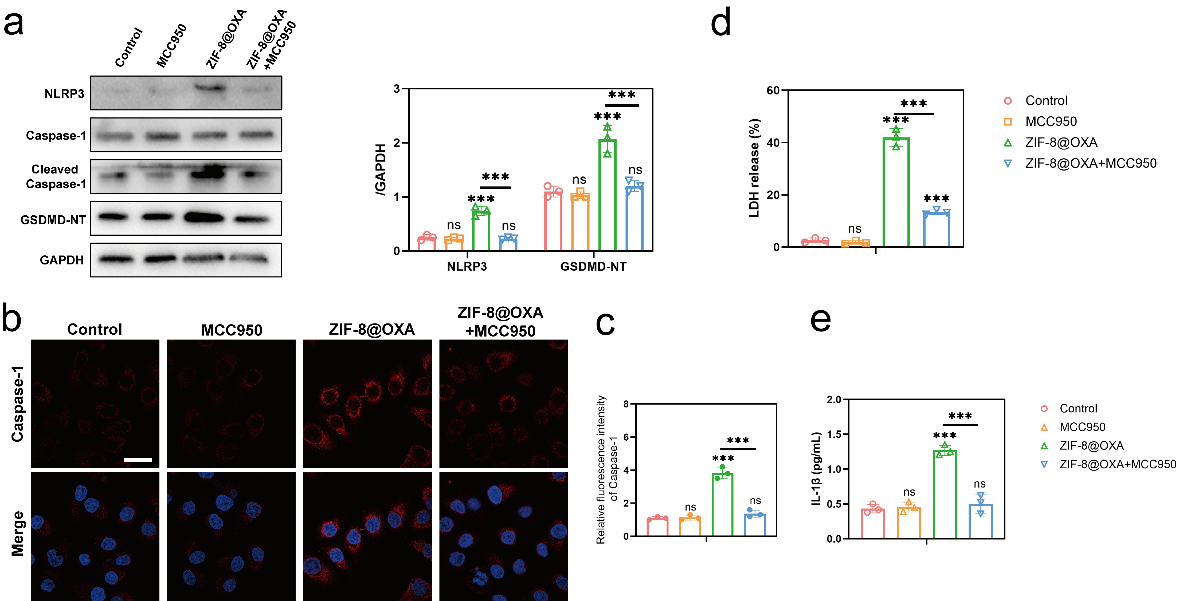


**Figure S25**. (a) Expression of NLRP3, Caspase-1, Cleaved Caspase-1, and GSDMD-NT expression in MC38 cells after treatment with and without MCC950 (n = 3). (b-c) Intracellular caspase-1 level and statistical analysis of treated MC38 cells (n = 3), scale bars:20 μm. (d-e) The relative amounts of released lactic dehydrogenase (LDH) and (e) the amounts of released intereukin-1β (IL-1β) (n = 3). * *p* < 0.05, ** *p* < 0.01, *** *p* < 0.001.


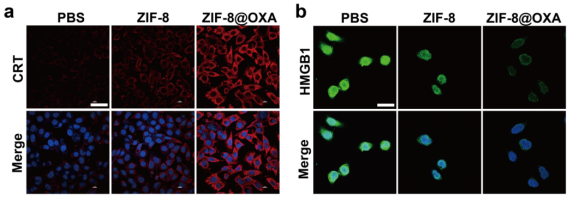


**Figure S26**. CLSM images of CRT and HMGB1 in treated MC38 cells (scale bar, 20 μm)


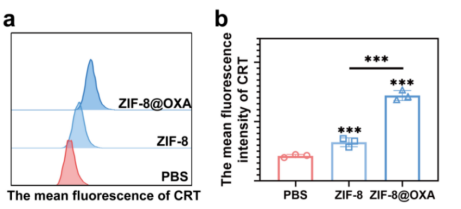


**Figure S27**. Flow cytometry analysis of CRT exposure on the cell surface of MC38 cells after various treatments (n = 3). * *p* < 0.05, ** *p* < 0.01, *** *p* < 0.001.


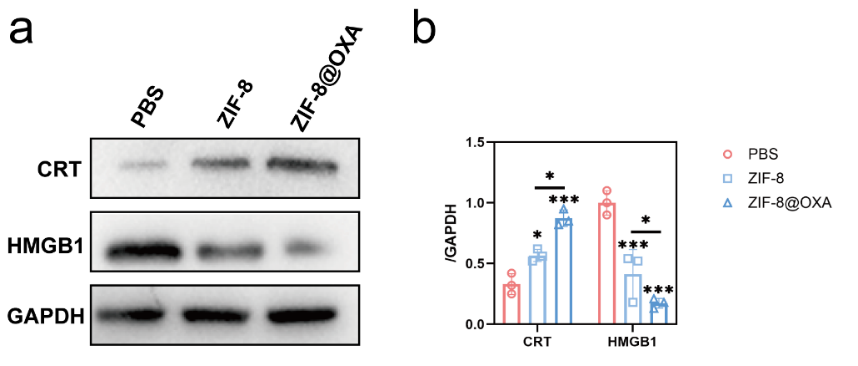


**Figure S28**. Expression of CRT and HMGB1 within MC38 cells after being incubated with ZIF-8@OXA NPs (n = 3). Data represent the mean ± SD (n = 3). * *p* < 0.05, ** *p* < 0.01, *** *p* < 0.001.


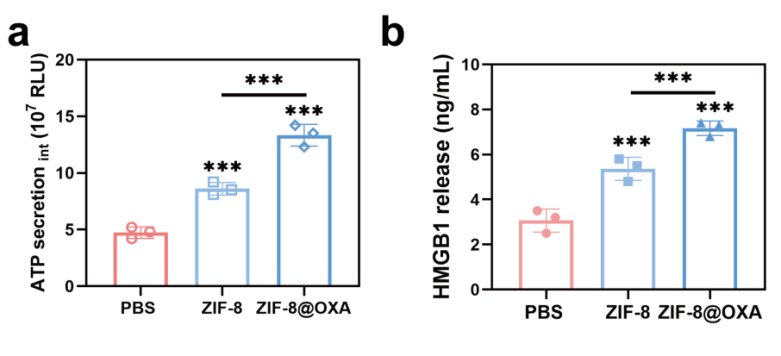


**Figure S29.** (a) ATP secretion detected by an enhanced ATP assay kit (n = 3). **(**b**)** HMGB1 released from MC38 cells detected by an enzyme-linked immunosorbent assay (ELISA) kit (n = 3). Data represent the mean ± SD (n = 3). * *p* < 0.05, ** *p* < 0.01, *** *p* < 0.001.


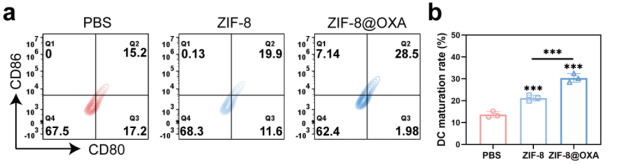
**Figure S30.** Flow cytometric images and statistical analysis for DCs maturation rates after various treatments (n = 3), * *p* < 0.05, ** *p* < 0.01, *** *p* < 0.001.

.
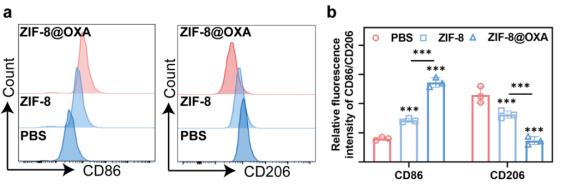


**Figure S31.** Flow cytometric images and statistical analysis for macrophages polarization rates after various treatments (n = 3), * *p* < 0.05, ** *p* < 0.01, *** *p* < 0.001.

.
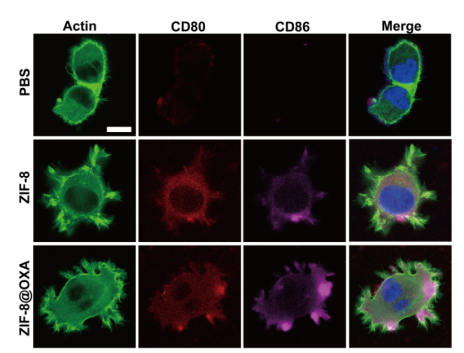


**Figure S32.** Representative immunofluorescence images of DC cells after treatment with ZIF-8@OXA NPs, stained for cell surface expression of β-actin (green), CD80 (red), CD86 (cy5), and nuclear (blue). Scale bar: 10 µm.


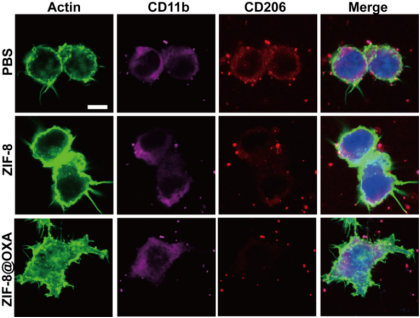


**Figure S33.** Representative immunofluorescence images of RAW 264.7 after treatment with ZIF-8@OXA NPs, stained for cell surface expression of β-actin (green), CD11b (cy5), CD86 (red), and nuclear (blue). Scale bar: 10 µm.


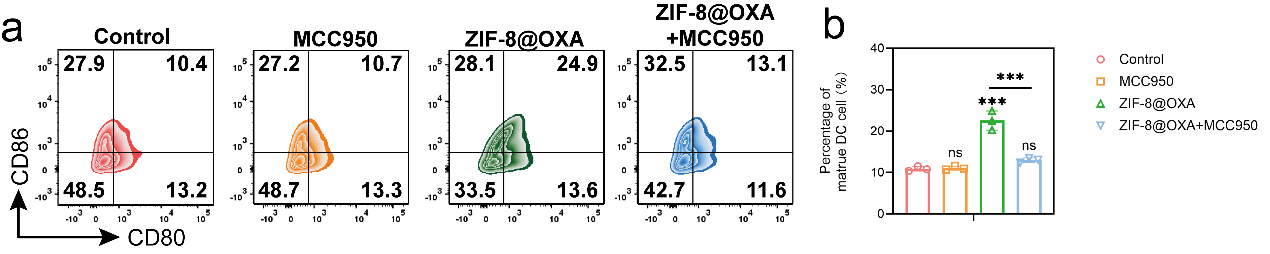


**Figure S34**. Flow cytometric images and statistical analysis for DCs maturation rates after various treatments in vitro (n = 3), * *p* < 0.05, ** *p* < 0.01, *** *p* < 0.001.


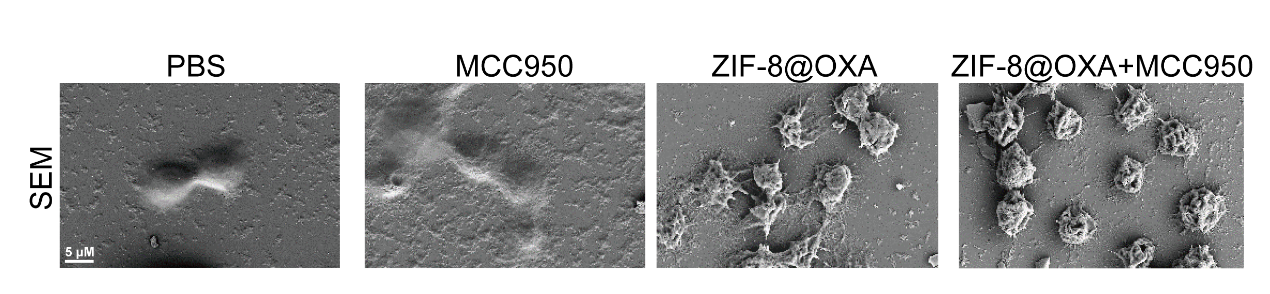


**Figure S35**. Bio-SEM of NETs (red boxes) in neutrophils after various treatments, scale bar: 5 μm.


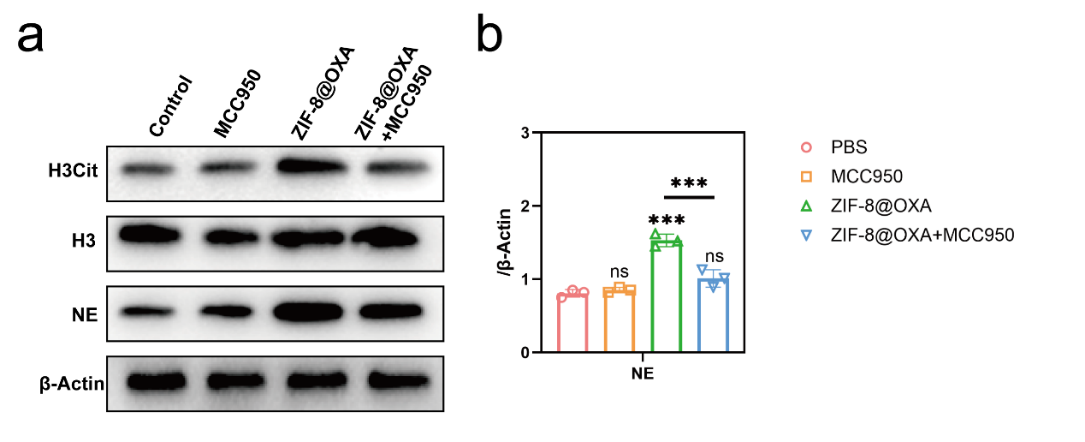


**Figure S36**. (a) The protein levels and (b) statistical analysis of H3Cit and NE were evaluated by Western blot (n = 3), * *p* < 0.05, ** *p* < 0.01, *** *p* < 0.001.


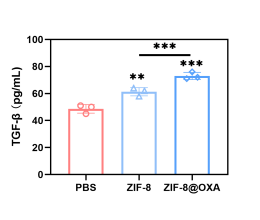


**Figure S37.** TGF-β in the supernatants were determined using ELISA assays (n = 3). * *p* < 0.05, ** *p* < 0.01, *** *p* < 0.001.


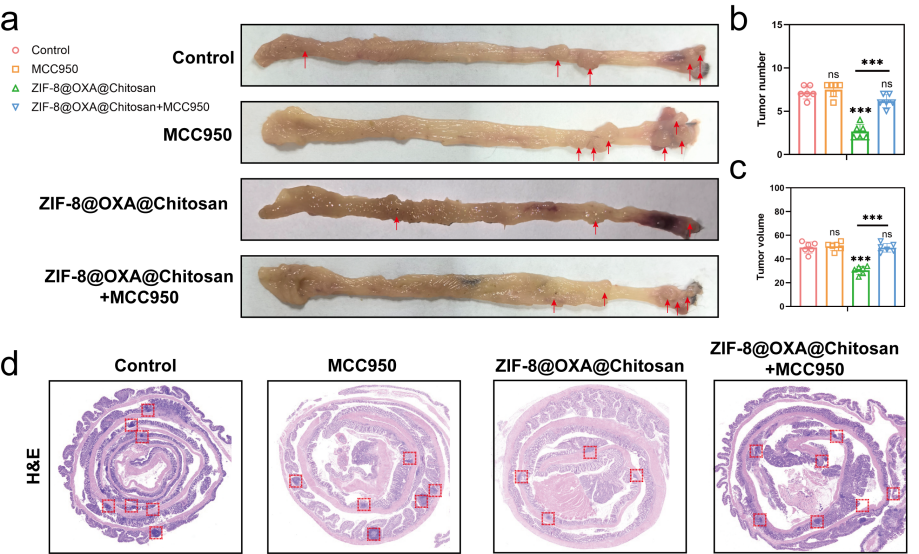


**Figure S38**. (a) Representative images of the colon after the different treatments. (b) Tumor number on day 18, when the mice were euthanized (n = 6). (c) Tumor volume on day 18 when the mice were euthanized. (d) H&E stained intestinal sections. * *p* < 0.05, ** *p* < 0.01, *** *p* < 0.001.


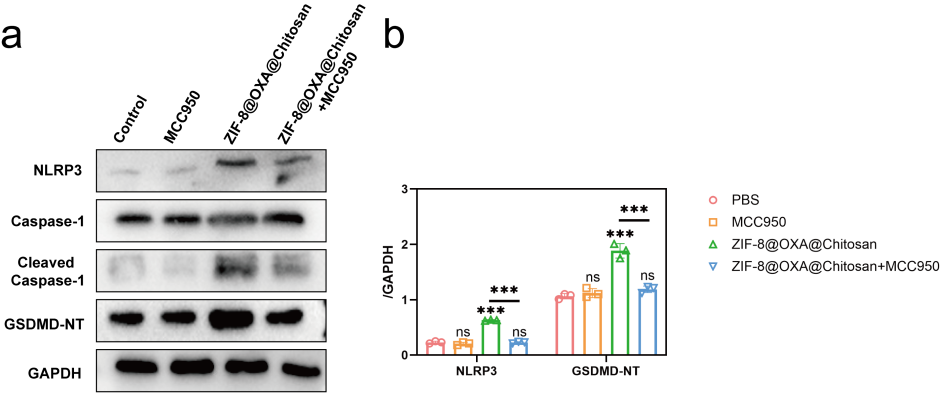


**Figure S39**. (a) Expression and (b) statistical analysis of NLRP3, Caspase-1, Cleaved Caspase-1, and GSDMD-NT expression in MC38 cells after treatment with and without MCC950 (n = 3). * *p* < 0.05, ** *p* < 0.01, *** *p* < 0.001.


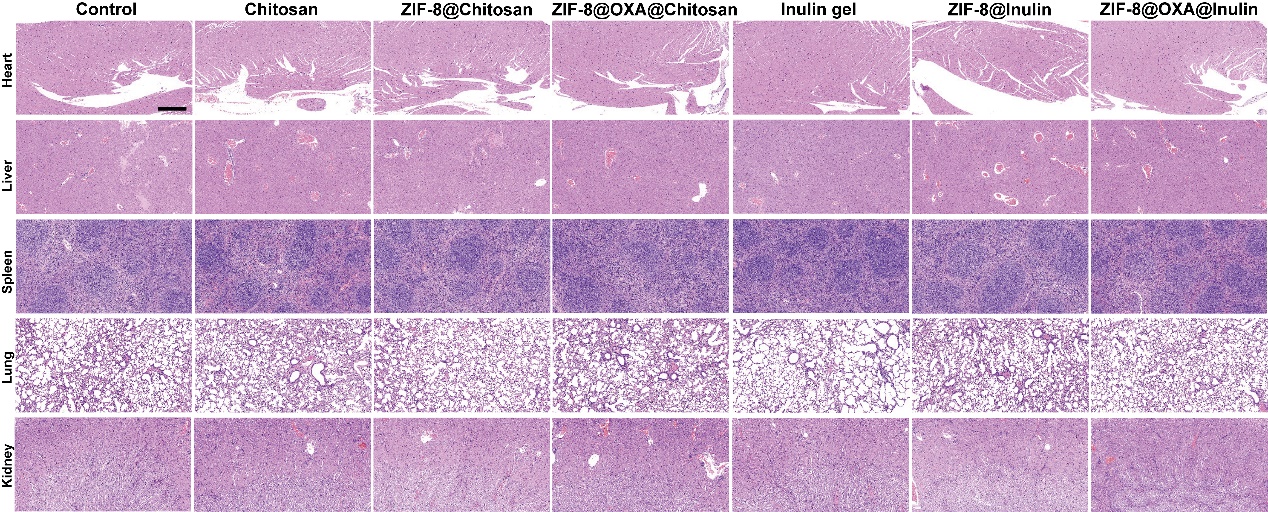


**Figure S40.** H&E staining images of major organs (lung, livers, spleens, kidneys, hearts) of bearing orthotopic colon cancer mice in control and treatment groups. Scale bar: 50 μm.


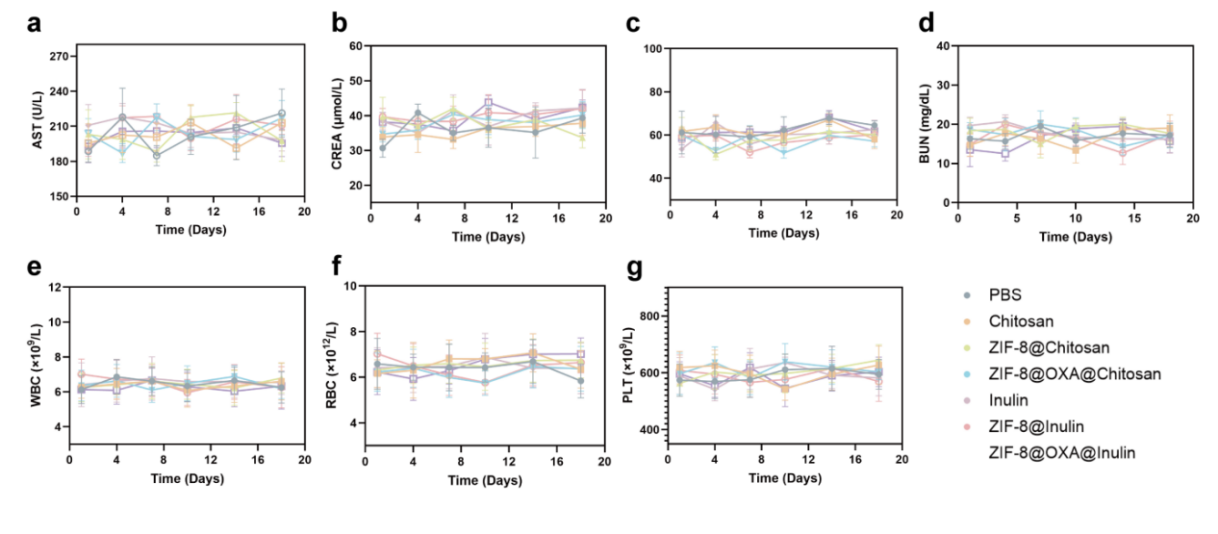
**Figure S41.** The detection of AST, CREA, ALT, BUN, WBC, RBC, and PLT after different treatment (n = 6).


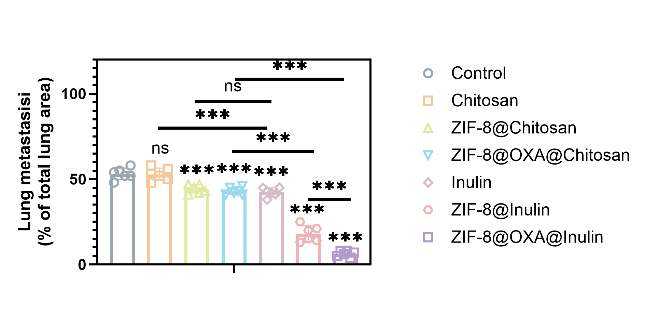


**Figure S42**. The number of metastatic foci (Figure 5k) in the lungs were quantified from H&E staining (n = 6). * *p* < 0.05, ** *p* < 0.01, *** *p* < 0.001.


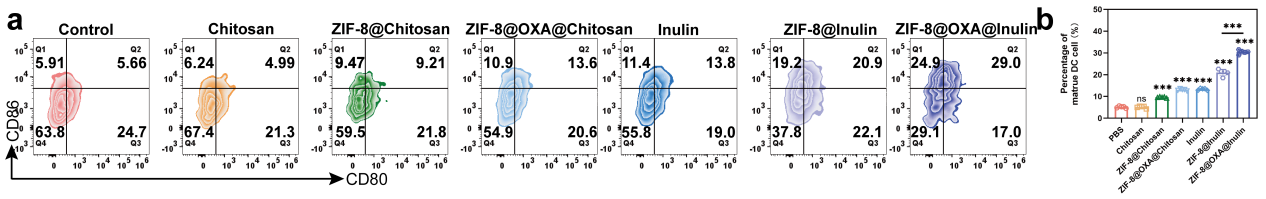


**Figure S43.** Flow cytometric images and statistical analysis for DCs maturation rates after various treatments (n = 6), * *p* < 0.05, ** *p* < 0.01, *** *p* < 0.001.


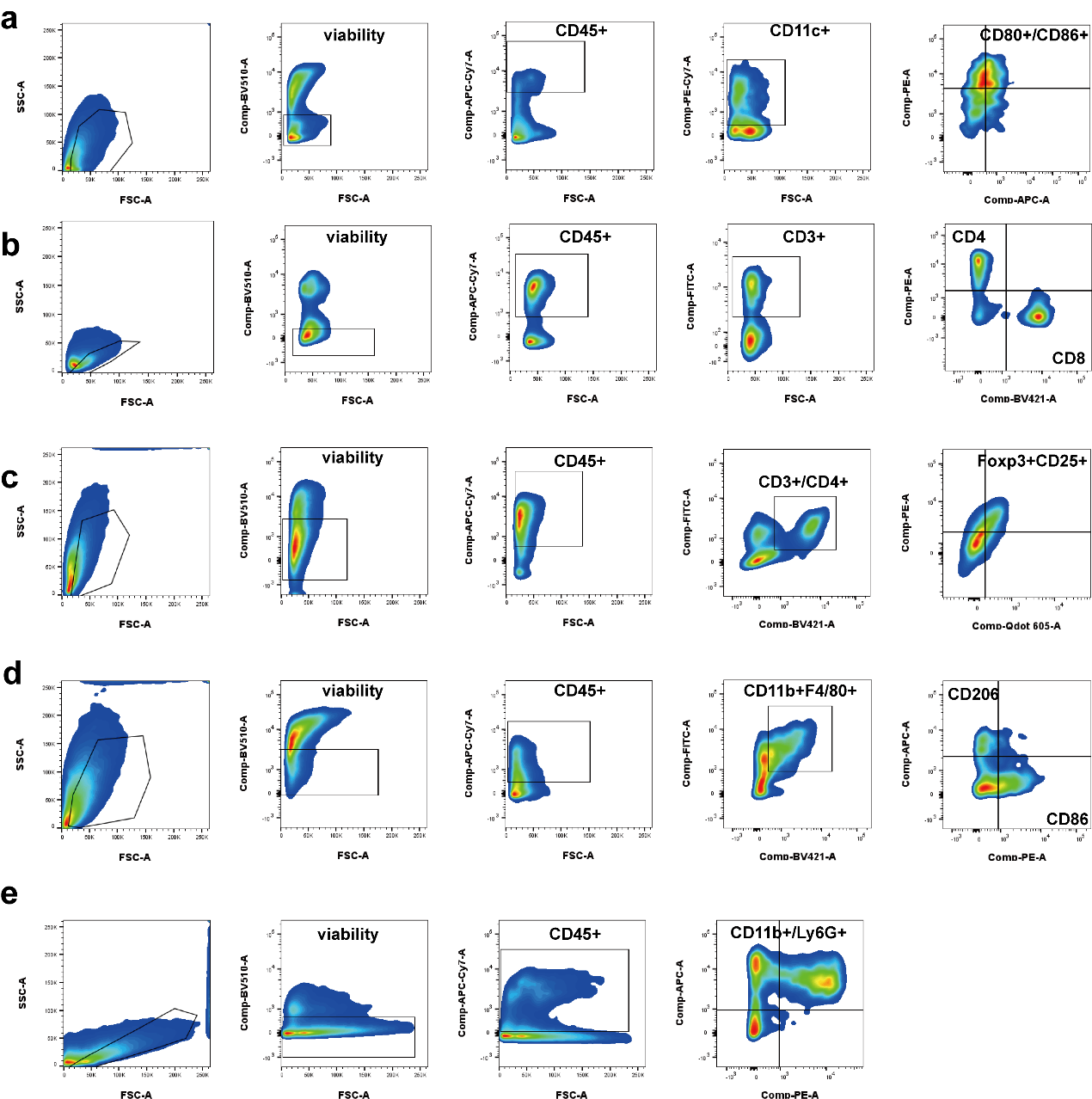


**Figure S44**. (a) The original data of flow cytometry results of the intratumor infiltration of DC maturation cells (Viability+CD45+CD11c+CD80+CD86+). (b) The original data of flow cytometry results of the intratumor infiltration of CD8+ T cells (Viability+CD45+CD3+CD8+ cells). (c) The original data of flow cytometry results of the intratumor infiltration of Tregs (Viability+CD45+CD3+CD4+Foxp3+ CD25+). (d) The original data of flow cytometry results of the intratumor infiltration of M1-like macrophages (Viability+CD45+CD11b+F4/80+CD80+) and M2-like macrophages (Viability+CD45+CD11b+ F4/80+CD206+). (e) The original data of flow cytometry results of the intratumor infiltration of neutrophils (Viability+ CD45+CD11b+Ly6G+).


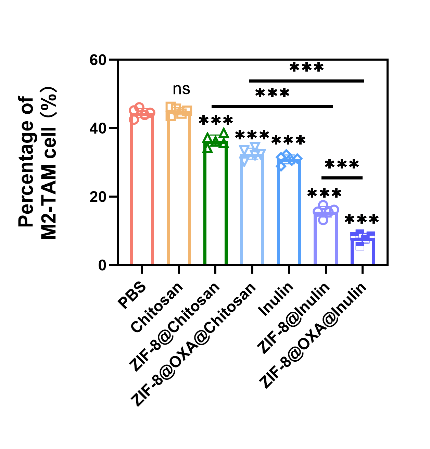


**Figure S45.** Statistical evaluation of the infiltration of M2-like TAMs after various treatments (n = 6), * *p* < 0.05, ** *p* < 0.01, *** *p* < 0.001.


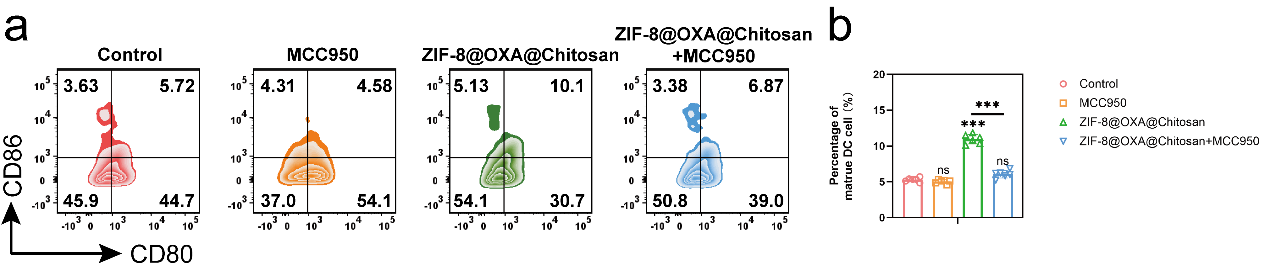


**Figure S46**. Flow cytometric images and statistical analysis for DCs maturation rates after various treatments in vivo (n = 6), * *p* < 0.05, ** *p* < 0.01, *** *p* < 0.001.


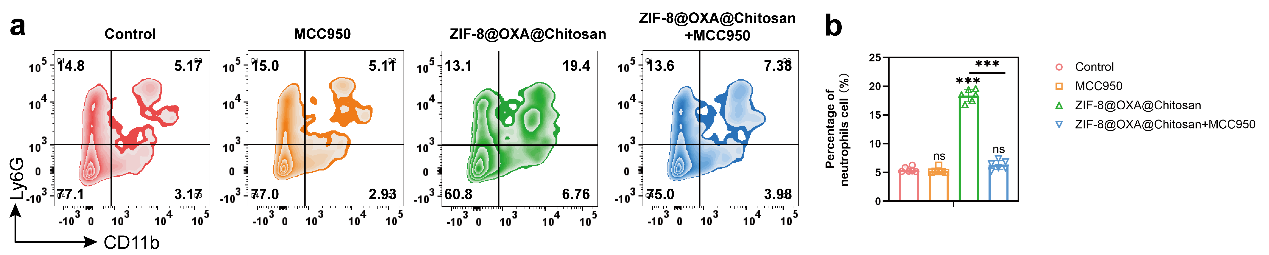


**Figure S47**. (a) Representative FCM analysis and (b) Statistical evaluation of neutrophils (CD45+CD11b+Ly6G+) infiltrating the orthotopic colon cancer after different treatments (n = 6). * *p* < 0.05, ** *p* < 0.01, *** *p* < 0.001.


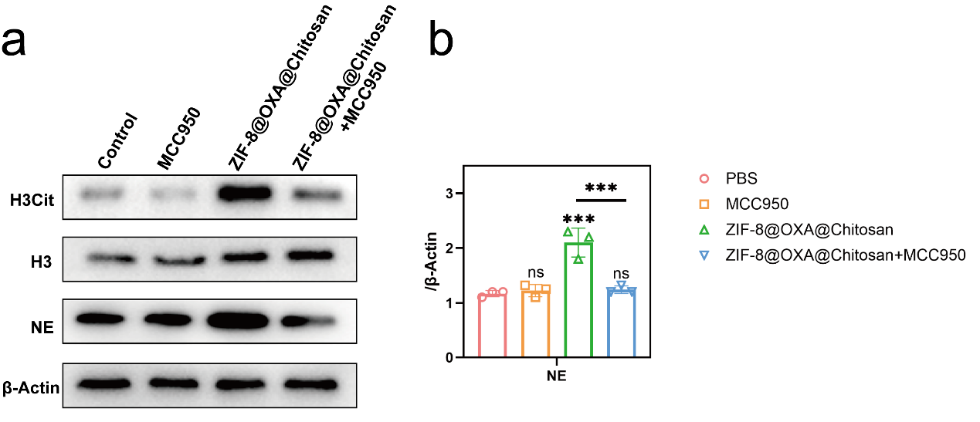


**Figure S48**. (a) The protein levels and (b) statistical analysis of H3Cit and NE were evaluated by Western blot (n = 3), * *p* < 0.05, ** *p* < 0.01, *** *p* < 0.001.


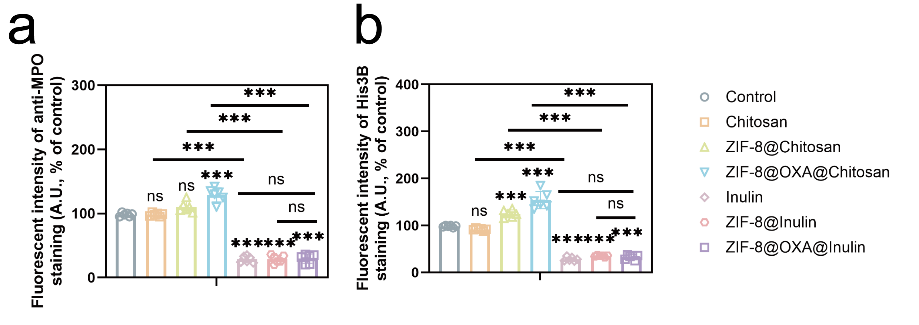


**Figure S49**. Quantification of images represented in (Figure 6i) from the colon of mice treated as indicated and stained for MPO (neutrophils) and cit-H3 (NETs) (n = 6). * *p* < 0.05, ** *p* < 0.01, *** *p* < 0.001.


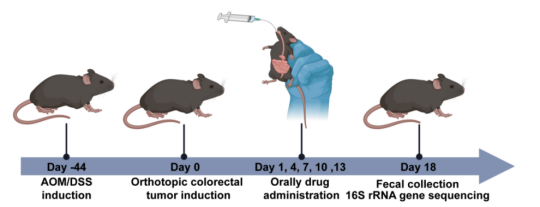


**Figure S50.** Treatment schedule for 16S ribosomal RNA gene sequencing after ZIF-8@OXA NPs-embedded inulin microspheres therapy on orthotopic colon tumor model (created using BioRender.com).


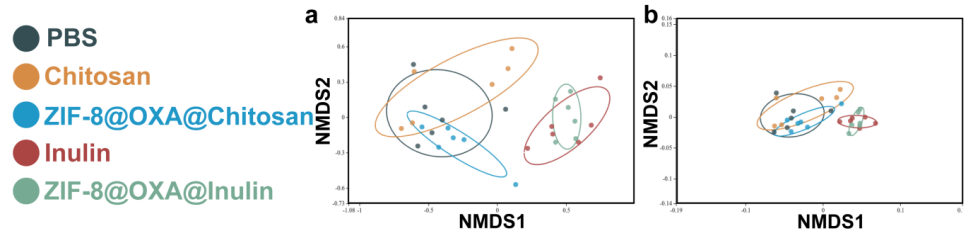


**Figure S51.** Microbial β-diversity NMDS analysis based on bray_curtis(a) and weighted_unifrac (b) distance at the OTU level at the end of the treatment (n = 6).


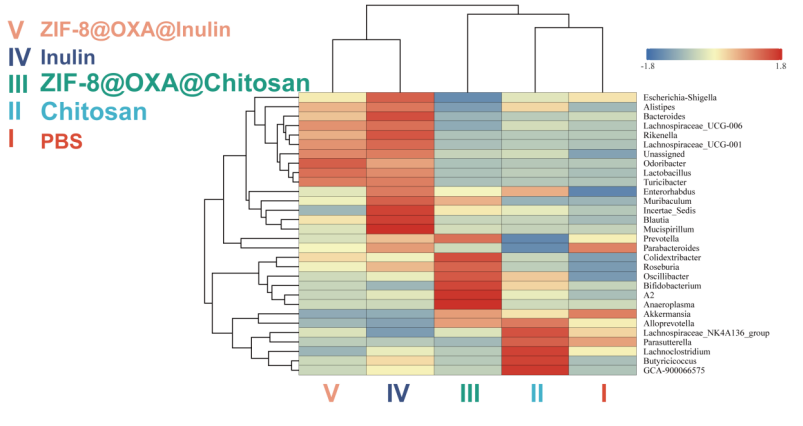


**Figure S52.** Heatmap showing relative abundance of the gut microbiota at the genus level.


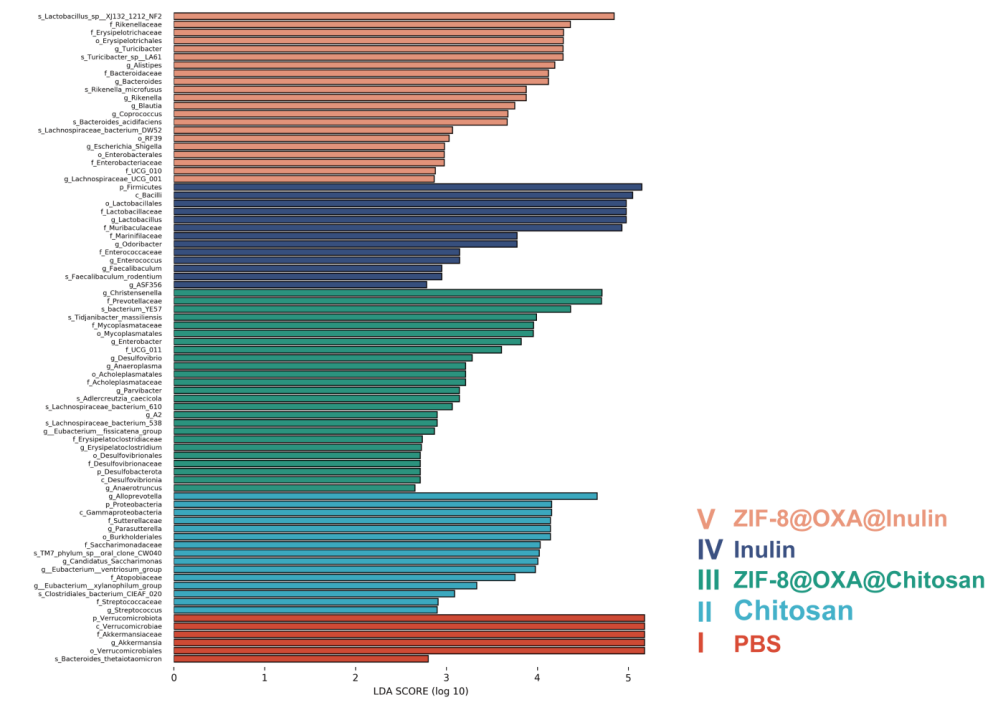


**Figure S53.** Linear discriminant analysis effect size (LefSe) analysis cladogram representing the inter-group significantly different taxas from Kingdom to OTU levels (LDA > 2, *p* < 0.05).


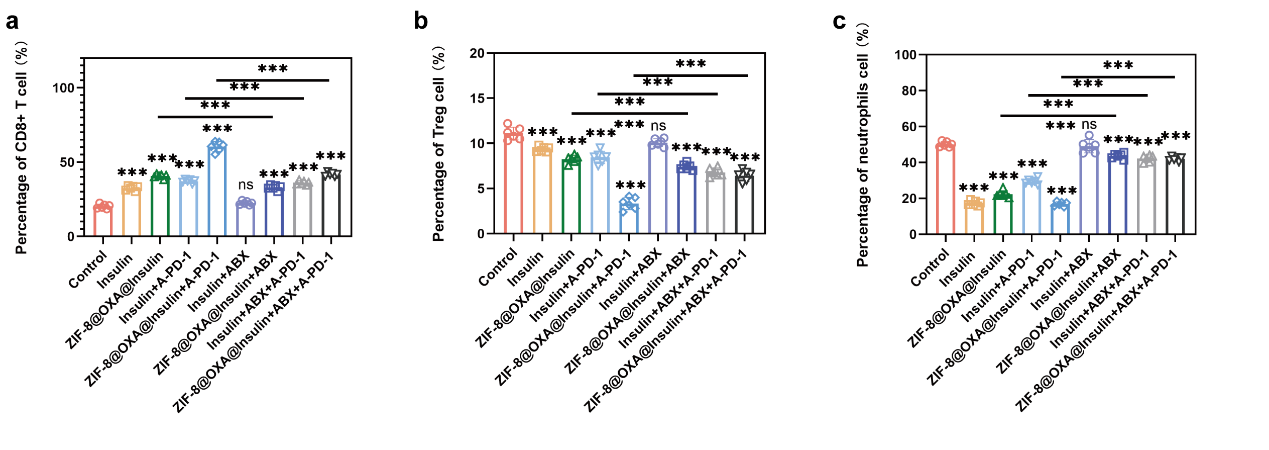


**Figure S54.** Statistical evaluation of the infiltration of CD8+ T cells, Treg cells and neutrophilis cells in orthotopic colon tumor (n = 6). * *p* < 0.05, ** *p* < 0.01, *** *p* < 0.001.


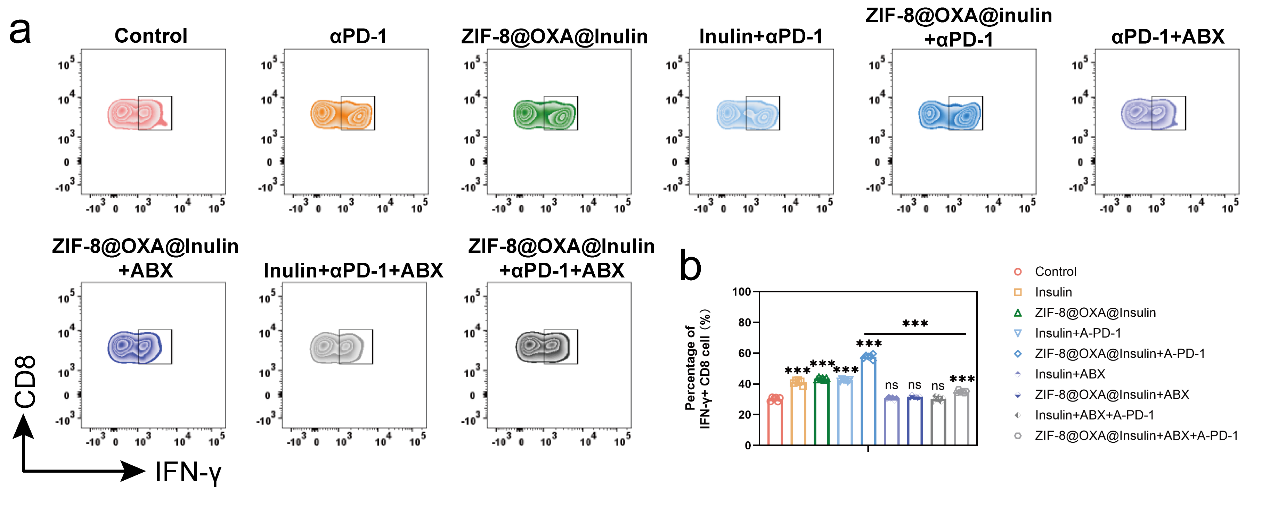


**Figure S55.** Flow cytometric images and statistical analysis for IFN-γ+CD8+ T cell rates after various treatments (n = 6), * *p* < 0.05, ** *p* < 0.01, *** *p* < 0.001.

**Supplementary Table 1.**

|  | **a** | **b** | **c** | **d** |
| --- | --- | --- | --- | --- |
| Particle size (μm) | 5.2 ± 0.5 | 10.4 ± 0.8 | 5.6 ± 0.6 | 11.2 ± 0.4 |
| Hydrodynamic size (μm) | 6.5 ± 0.5 | 14.2 ± 0.7 | 6.3 ± 0.9 | 15.4 ± 0.7 |
| Zeta potential (mV) | -28.5 ± 4.2 | -29.5 ± 5.5 | -30.0 ± 2.5 | -31.5 ± 3.4 |
| Drug loading (%) | 10.2 ± 2.5 | 8.3 ± 2.1 | 11.3 ± 1.8 | 9.5 ± 1.3 |
| Encapsulation efficiency (%) | 45.3 ± 3.6 | 40.5 ± 2.5 | 48.2 ± 3.5 | 43.2 ± 2.5 |

**Table 1**. Characterization of microsphere formulations. (a) ZIF-8@OXA@inulin, (b) ZIF-8@OXA@inulin@CAP, (c) ZIF-8@OXA@chitosan, and (d) ZIF-8@OXA@chitosan@CAP. Data are mean ± SD (n = 3). Planned pairwise comparisons were restricted to (a) vs (c) and (b) vs (d). Symbols on (a) indicate significance vs (c); symbols on (b) indicate significance vs (d): * *p* < 0.05, ** *p* < 0.01, *** *p* < 0.001. Unmarked values are not significant.
